# Illustration of the variation in the content of flavanone rutinosides in various citrus germplasms from genetic and enzymatic perspectives

**DOI:** 10.1093/hr/uhab017

**Published:** 2022-01-18

**Authors:** Wenyun Li, Gu Li, Ziyu Yuan, Mingyue Li, Xiuxin Deng, Meilian Tan, Yuhua Ma, Jiajing Chen, Juan Xu

**Affiliations:** 1 Key Laboratory of Horticultural Plant Biology (Ministry of Education), College of Horticulture and Forestry, Huazhong Agricultural University, No.1 Shizishan Street, Hongshan District, Wuhan 430070, China; 2 Guizhou Fruit Institute, Guizhou Academy of Agricultural Sciences, No.1 Jinnong Road, Huaxi District, Guiyang 550006, China; 3 The Oil Crops Research Institute of the Chinese Academy of Agricultural Sciences, No.2 Xudong Second Road, Wuchang District, Wuhan 430062, China

## Abstract

In citrus, 1,6-rhamnosytransferase (1,6RhaT) and 1,2-rhamnosytransferase (1,2RhaT) catalyze flavanone-7-*O*-glucosides to form nonbitter flavanone rutinosides (FRs) and bitter flavanone neohesperidosides (FNs), respectively. As revealed in this study of fruit peels from 36 citrus accessions, FRs varied from undetectable levels in pummelo and kumquat to being the dominant flavonoids in sweet orange and loose-skin mandarins. Furthermore, a previously annotated full-length *1,6RhaT-like* gene was identified as another 1,6RhaT-encoding gene by *in vitro* experiments. In total, 28 alleles of full-length *1,6RhaTs* were isolated and classified into A, B and C types with only type A alleles encoding a functional protein. Coincidently, only the accessions that contained FRs harbored type A alleles, as was further verified in two F1 hybrid populations. Moreover, the inferior substrate conversion efficiency of 1,6RhaTs in comparison with that of 1,2RhaT *in vitro* might partly explain the lower proportions of FRs to total flavanone disaccharides in citrus hybrids harboring both functional rhamnosyltransferases. Our findings provide a better understanding of FR content variations among citrus and are meaningful for a mechanistic illustration of citrus flavonoid metabolism and fruit quality improvement practices.

## Introduction

As the most abundant polyphenol compounds in citrus, flavonoids comprise a large family that includes flavanones, flavonols, flavones, isoflavones, anthocyanidins, and flavanols [1, 2]. Flavonoids reportedly possess a wide range of biological activities, including antioxidant, anticancer, antimicrobial, anti-inflammatory, anti-obesity and cardiovascular and cerebrovascular disease-preventing activities [3–6]. Most flavonoids in citrus are present in the form of glycosyl derivatives, which increases their stability, solubility and bioavailability [7]. Flavanone rutinosides (FRs) and flavanone neohesperidosides (FNs), which are the predominant flavanone disaccharides in citrus, contain the α-L-rhamnosyl-β-D-glucoside disaccharide moiety bound to the flavanone aglycone and exhibit different glycosidic links (β1 → 2 of FRs, β1 → 6 of FNs) between the two sugar units. Recently, FRs have received increasing attention due to their wide range of features that are beneficial to human health [8–10]. For example, hesperidin is the main pharmacological component of Pericarpium Citri Reticulatae (chenpi), which can effectively relieve skin inflammation, muscle pain, coughing and breath difficulties [11].

Flavanone disaccharides are synthesized *via* the phenylpropanoid pathway in plants as stabilized end products resulting from two glycosylation steps [12]. The first glycosylation step involves the reaction of flavanone aglycones to flavanone-7-*O-*glucosides catalyzed by 7-*O-*glucosyltransferase (7-GlcT), and these products are further converted to flavanone disaccharides of FRs and FNs by 1,6-rhamnosytransferase (1,6RhaT) and 1,2-rhamnosytransferase (1,2RhaT), respectively [13] (Supporting Fig. S1). These two flavanone disaccharides contribute significantly to the flavor of citrus fruits by providing nonbitter FRs (eriocitrin, didymin, narirutin and hesperidin, *etc*.) and bitter FNs (neoeriocitrin, poncirin, neohesperidin and naringin, *etc*.), respectively [14]. It is worth noting that FRs and FNs are synthesized from the same substrate, and competition may exist within germplasms harboring both functional genes.

A considerable number of studies have investigated the specific accumulation of FRs and FNs in citrus germplasms and the changes in the contents of these compounds among different developmental stages or tissues [15–18]. High levels of FRs are present in orange, mandarin and lemon; low levels of FRs are present in grapefruit and bitter orange, which contain high levels of FNs; and FRs were not detected in pummelo [19]. The available evidence shows that the accumulation of FRs varies among citrus germplasms, but the mechanisms are not well understood. Fortunately, based on our previous elucidation of the molecular mechanism underlining the variations in the FN profiles among different citrus germplasms [20], revealing the mechanisms underlying the variations in the FR profiles in different citrus germplasms is feasible.

In this study, the FRs in various citrus accessions covering the main cultivated species, wild species and their relatives were investigated. Another *1,6RhaT* gene was identified, which implies that there are multiple *1,6RhaT* genes in citrus. The *1,6RhaT* genes and their allelic variations were then characterized *in vitro*. Furthermore, the production of FNs and FRs in two hybrid cross populations harboring both functional *1,2RhaT* and *1,6RhaT* genes was further investigated. This study will contribute to a better understanding of the accumulation of flavanone disaccharides with different bioactive characteristics in various citrus germplasms and is thus meaningful for improving fruit quality.

## Materials and methods

### Plant materials and compounds

Thirty-four accessions were obtained from the National Citrus Breeding Centre (NCBC) at Huazhong Agricultural University, Wuhan, China, and two other accessions were obtained from Haohuahong Town, Huishui County, Guizhou Province, China (Table S1). For each accession, young leaves and 15–18 fruits were randomly collected from at least three healthy trees at their respective commercial maturation stages at approximately 230 d post anthesis (DPA).

Hybrid populations of “Hirado Butun” (HB) pummelo (*C. maxima*) × Fairchild tangelo (*Citrus reticulata* × *Chrysopelea paradisi*) and red tangerine (*Citrus reticulata*) × Trifoliata orange (*Poncirus trifoliata*) were established in 2003, and fruits and leaves from 37 and 66 F_1_ plants were collected from these two populations, respectively.

All fruits and leaves were randomly divided into three biological replications. The fruit peel (flavedo and albedo) tissues were immediately separated, frozen in liquid nitrogen, ground into a fine powder and stored at −80°C. All operations were performed under dim light conditions, and long-term exposure to open air was avoided to prevent the degradation of flavonoids.

Transgenic tobacco bright yellow (BY2) calli harboring Cm1,2RhaT were obtained as described by Chen et al. (2019).

Authentic standard compounds of UDP-glucose, poncirin, didymin, neoeriocitrin, eriocitrin, naringin, narirutin, neohesperidin, hesperidin, naringenin, hesperetin, naringenin-7-*O*-glucoside, and hesperetin-7-*O*-glucoside were purchased from Shanghai Yuanye Bio-Technology Co., Ltd. (Shanghai, China), with a high purity of 98%. UDP-rhamnose was purchased from Angfei Biological Technology Co., Ltd. (Guangdong, China).

### Extraction of flavonoids and determination of FRs and FNs

The extraction and separation of the flavanone disaccharides were conducted as described previously [17]. Flavonoids were separated using a gradient elution program with a flow rate of 1 ml/min, a column temperature of 35°C and a Waters 1525 binary system equipped with a 2996 photodiode array detector, a 717 Plus autosampler (Waters Corp., Milford, MA, USA) and a C18 column (250 mm × 4.6 mm, 5 μm, Thermo Scientific, Waltham, MA, USA). Subsequently, 0.15% formic acid diluted in water was prepared as mobile phase A, and 0.15% formic acid-acetonitrile was prepared as mobile phase B. The data were analyzed using Empower Chromatography Manager software (Waters Co., Milford, MA, USA).

### RNA isolations, cDNA synthesis and real-time PCR

Total RNA was isolated using an EASYspin Plus Plant RNA Kit (Aidlab Biotechnologies Co., Ltd.). HiScript II Reverse Transcriptase (Aidlab Biotechnologies Co., Ltd.) was used for the amplification of cDNA. qRT-PCR was performed using a Prism 7900HT (Applied Biosystems) with the SYBR Green system (Hieff qPCR SYBR Green Master Mix, Yeasen Biotech Co., Ltd.). All gene expression analyses were performed with three independent biological replicates. The primers used in the analysis are listed in Supporting Table S2. The *actin* gene was used as the reference control.

### BLAST analysis, phylogenetic analysis and gene cloning

BLAST analysis was performed against the Phytozome 12 database (https://phytozome.jgi.doe.gov/pz/portal.html#!search?show=BLAST) and *Citrus sinensis* Genome Annotation Project database (http://citrus.hzau.edu.cn/cgi-bin/orange/blast) using *Cs1,6RhaT* (accession number: DQ119035) as the reference sequence. ClustalW (http://www.genome.jp/tools-bin/clustalw) was utilized for multiple alignments of the nucleotide sequences of the alleles in 36 accessions. Phylogenetic analysis was generated using MEGA 7.0 according to the maximum likelihood method. There were 1000 bootstrap replications to test the reliability.

Young leaves of 36 accessions and two F1 hybrid populations were collected for genomic DNA extraction according to the protocol described by Cheng et al. [21] To amplify the complete coding sequence of *1,6RhaT* genes from both gDNA and cDNA templates, specific PCR primers were designed based on *Cs1,6RhaT* (Supporting Table S2). The primers were synthesized by Sangon Biotech Co., Ltd. (Shanghai, China). To ensure accuracy, at least eight positive clones of each accession were sequenced by TSINGKE Biological Technology Co., Ltd. (Beijing, China).

### Expression and biotransformation assay in tobacco BY2 cells

Seven *1,6RhaT* alleles that were found at a higher frequency (A1, A5, A10, B2 and C4) during isolation or appeared in some unique accessions (A8 and C6) were cloned into the pH7WG2D vector under the control of a CAMV-35S promoter harboring green fluorescent protein (GFP) and transferred into *Agrobacterium* GV3101. The resulting construct was used to stably transform BY_2_ callus suspensions according to a published protocol [20, 22]. The transgenic calluses were confirmed by fluorescence detection through epifluorescence stereomicroscopy at 488 nm, PCR amplification and sequencing. The positively transformed calli were subjected to subsequent substrate feeding analysis.

To carry out the biotransformation assays, a substrate feeding experiment using BY2 cells was performed according to the protocol described by Frydman et al. [13]. Briefly, transgenic and wild-type BY_2_ calli were suspended in Erlenmeyer flasks for one week at 28°C with shaking at 140 rpm. Subsequently, 1 g of callus was diluted in 20 mL of MS liquid media and incubated under the same conditions for 4 d. Flavonoid substrates (mg/ml) were added to the flask and then incubated for another 2 d. The resulting BY_2_ cells were harvested and used for extraction and determination of flavonoids.

### Protein expression and purification

The expression and purification of *Cme1,6RhaT-like* was performed as described by Peng et al. [23]. In brief, *Cme1,6RhaT-like* was cloned into the pMal-c2x expression vector with a maltose-binding protein tag. Recombinant proteins were expressed in BL21 (DE3) cells and induced with IPTG at 16°C. The harvested cells were lysed in phosphate-buffered saline pH 7.4 containing 400 mM NaCl by sonication at 4°C. The protein was transferred into a column containing dextrin beads 6FF and purified by washing buffer (20 mM Tris–HCl, 20 mM NaCl, 1 mM EDTA and 1 mM DTT, pH 7.4) and elution buffer (20 mM Tris–HCl, 1 mM EDTA, 10 mM maltose, and 1 mM DTT, pH 7.4).

*Cp1,2RhaT and Cp1,6RhaT* were expressed and purified following a similar protocol.

### Assay enzyme activity and kinetics

The enzyme assay was performed according to Peng et al. [23]. The glycosylation reaction was performed in a total volume of 100 μl containing 5 μg of purified glycosyltransferases, 5 μg of flavonoid substrates, 5 μg of UDP-rhamnose, and 0.25 mM MgCl_2_ in phosphate buffered saline (50 mM, pH 7.4). The reaction was then incubated for 30 min at 35°C. The reaction was stopped by the addition of 300 μl of ice-cold methanol, and the resulting products were subjected to LC–MS analysis.

To determine the enzymatic kinetic constants of Cp1,2RhaT and Cp1,6RhaT, their activities were determined using 10 to 400 μM flavonoid aglycones with 5 μg UDP-rhamnose. All kinetic parameters were calculated using the Michaelis–Menten model (SigmaPlot, version 12.5).

## Results

### FR accumulation is species-dependent in various citrus germplasms

In our previous study, flavonoids were found to accumulate at higher levels in fruit peel (flavedo and albedo) than in the pulp of most citrus germplasms. Consequently, substantial variations in the contents of FRs and FNs were observed among the fruit peels of 36 accessions (Supplement Table S1). These accessions cover the main cultivated species, wild species and their relatives, including trifoliata orange (Poncirus trifoliata), kumquat (*Citrus japonica*), loose-skin mandarin (Citrus reticulata), sweet orange (Citrus sinensis), pummelo (Citrus maxima), grapefruit (*Citrus paradisi*), lemon (Citrus limon), citron (Citrus medica) and ichang papeda (*Citrus ichangensis*).

**Figure 1:**
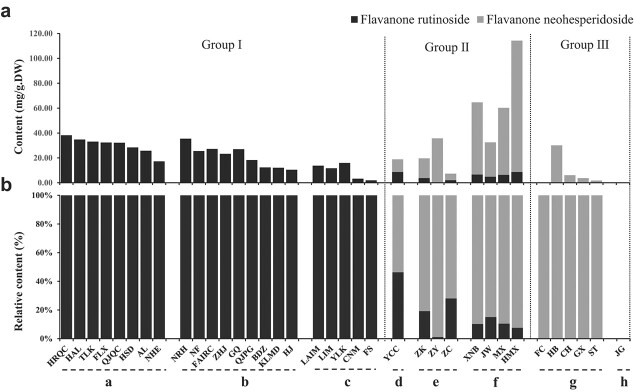
Percentages and contents of FNs and FRs in 36 citrus accessions. The black color on the column chart represents FR, and the gray color represents FN. (a) Contents of FNs and FRs (mg/g DW) in 36 citrus accessions. (b) Percentages (%) of FNs and FRs in 36 citrus accessions. The letters a to h indicate accession collections of orange, loose-skin mandarin, lemon and citrons, ichang papeda, trifoliate orange, grapefruit, pummelo and kumquat, respectively. The abbreviations of the 36 citrus accessions are shown in Supplementary Table S1.

These citrus accessions could be divided into three groups according to their contents of FRs (Fig. 1). The accessions in Group 1, such as sweet orange, loose-skin mandarin and citrons, had high levels of FRs but undetectable levels of FNs. Group 2 included accessions with both FRs and FNs, such as ichang papeda, trifoliate orange and grapefruit. However, the proportions of FR among the total flavanone disaccharides were lower than those of FNs. Group 3 was comprised of pummelo and kumquat and exhibited undetectable levels of FRs; moreover, pummelo was enriched in FNs, whereas FNs were undetectable in kumquat. These results showed that the citrus FR profiles are species-dependent. The associations among the contents and proportions of the FRs and the genetic background in various citrus accessions was then investigated.

**Figure 2:**
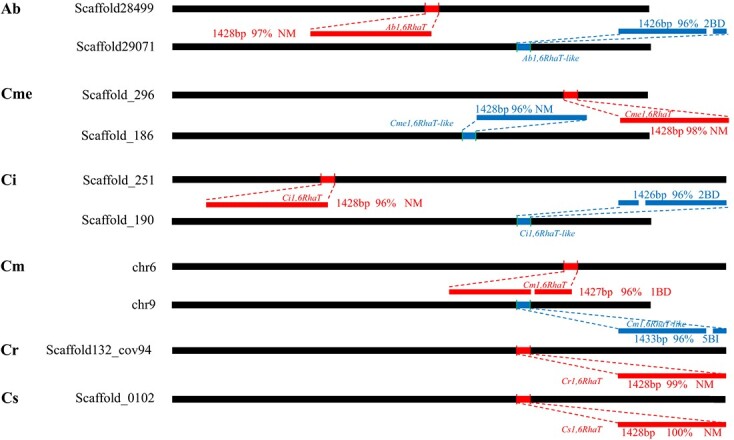
Schematic view of chromosomal loci of Cit1,6RhaT and 1,6RhaT-like in six citrus species. The percentage values indicate the nucleotide sequence homology to Cs1,6RhaT. The red segments represent Cit1,6RhaT; the blue color represents Cit1,6RhaT-like. NM, normal sequence; BD, base deletion; BI, base insertion; Cm, Citrus maxima; Ci, C. ichangensis; Cs, C. sinensis; Cr, C. reticulata; Cme, C. medica; Ab, Atalantia buxifolia.

### Identification of a new *1,6RhaT* gene in citrus genomes

A *1,6RhaT-like* gene with high nucleotide sequence homology to *Cs1,6RhaT* (GenBank accession No. AY048882) was identified in the genomes of various citrus species (Fig. 2). The *1,6RhaT-like* gene was annotated in the genomes of various citrus species with the exception of sweet orange and loose-skin mandarin and it was located on different loci from those of *1,6RhaT*. The genomic sequences revealed that *1,6RhaT-like* shared high nucleotide sequence identities (approximately 96%) with *Cs1,6RhaT*, and various base deletions and insertions were found in Atalantia buxifolia, C. maxima and *C. ichangensis*, whereas an intact *1,6RhaT-like* was only found in *Citrus medica* with an ORF of 1428 bp.

**Figure 3:**
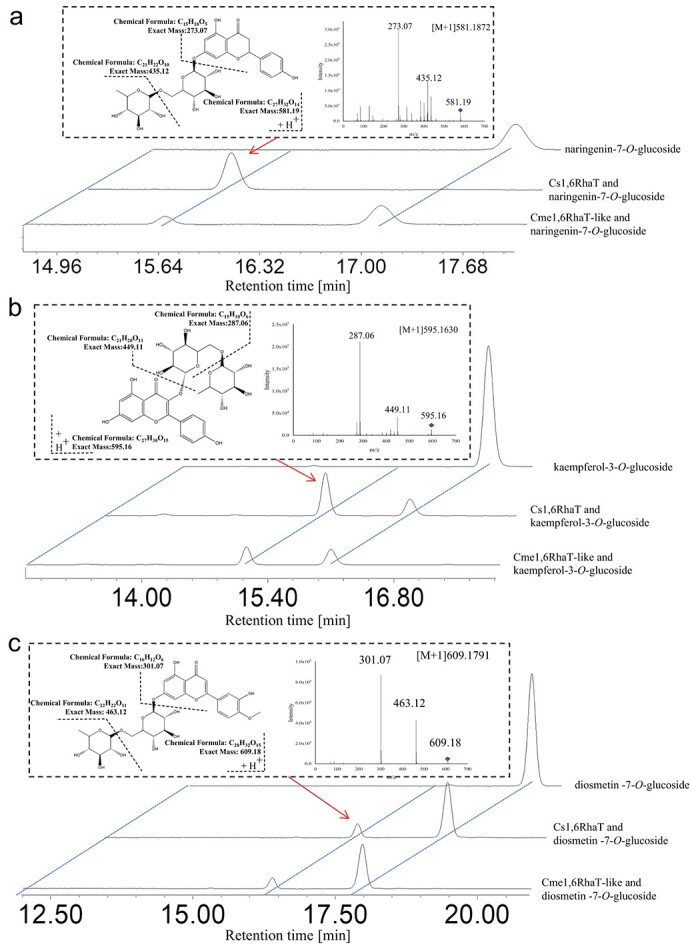
HPLC chromatogram of reaction products of recombinant Cme16RhaT-like and Cs1,6RhaT and MS2 spectra of the products. (a) Naringenin-7-O-glucoside was used as the substrate. (b) Kaempferol-3-O-glucoside was used as the substrate. (c) Diosmetin-7-O-glucoside was used as the substrate.

*Cme1,6RhaT-like* from Eureka lemon was isolated, sequenced and subjected to an analysis of its catalytic activity. *Cme*1,6RhaT-like was expressed in *Escherichia coli* and characterized. After the application of naringenin-7-*O*-glucoside and UDP-rhamnose as substrates, a new peak was detected and identified as narirutin by comparing the retention time and the MS2 spectra of the corresponding product of Cs1,6RhaT (Fig. 3a). To ensure its catalytic activity, we used other types of flavonoids, including flavone (kaempferol-3-*O*-glucoside) and flavonol (diosmetin-7-*O*-glucoside), as substrates (Fig. 3b-c). The results showed that Cme1,6RhaT-like also exhibited rhamnosylation activity toward these two types of flavonoids and converted these substrates into their corresponding flavanone disaccharides.

Thus, our results showed that *Cme1,6RhaT-like* is another *1,6RhaT* gene, and multiple *1,6RhaT* genes are found in citrus. Consequently, we defined *1,6RhaT* and *1,6RhaT-like* as *1,6RhaTs* in the rest of the manuscript.

**Table 1:** 1,6RhaT alleles related to FR phenotypes in 36 citrus accessions

Samples	Abbreviation	Alleles isolated	Length (bp)	Scientific name	Deduced amino acid (aa)	Phenotype
“Cara Cara” navel orange	HRQC	A1, A10	1428	*Citrus sinensis*	Intact protein (475aa)	+
Hong Anliu sweet orange	HAL	A1, A10	1428	*Citrus sinensis*	Intact protein (475aa)	+
Tarocco blood orange	TLK	A1, A10	1428	*Citrus sinensis*	Intact protein (475aa)	+
Valencia orange	FLX	A1, A10	1428	*Citrus sinensis*	Intact protein (475aa)	+
Seike navel range	QJQC	A1, A10	1428	*Citrus sinensis*	Intact protein (475aa)	+
Washington navel orange	HSD	A3, A10	1428	*Citrus sinensis*	Intact protein (475aa)	+
Anliu sweet orange	AL	A1, A10	1428	*Citrus sinensis*	Intact protein (475aa)	+
Newhall navel orange	NHE	A1, A10	1428	*Citrus sinensis*	Intact protein (475aa)	+
Niu Rouhong tangerine	NRH	A5, A10	1428	*Citrus sinensis*	Intact protein (475aa)	+
Nanfengmiju tangerine	NF	A1, A2	1428	*Citrus sinensis*	Intact protein (475aa)	+
Fairchild tangelo	FAIRC	A5, A10	1428	*Citrus reticulata × Citrus* *paradisi*	Intact protein (475aa)	+
Zhu Hongju tangerine	ZHJ	A5, A10	1428	*Citrus reticulata*	Intact protein (475aa)	+
“Guoqing No.1” satsuma mandarin	GQ	A1	1428	*Citrus reticulata*	Intact protein (475aa)	+
Qingjiangponkan mandarin	QJPG	A1, A10	1428	*Citrus reticulata*	Intact protein (475aa)	+
Bendizao tangerine	BDZ	A6	1428	*Citrus reticulata*	Intact protein (475aa)	+
Clementine	KLMD	A5, A10	1428	*Citrus reticulata*	Intact protein (475aa)	+
Red tangerine	HJ	A4, A10	1428	*Citrus reticulata*	Intact protein (475aa)	+
Lime	LAIM	A1, A17	1428	*Citrus aurantiifolia*	Intact protein (475aa)	+
Limonia	LIM	A12, A18	1428	*Citrus limonia*	Intact protein (475aa)	+
Eureka lemon	YLK	A11, A16	1428	*Citrus limon*	Intact protein (475aa)	+
Rough lemon	CNM	A16, B3	1428/1433	*Citrus jambhiri*	Intact protein (475aa)/Truncated protein (174aa)	+
Fingered citron	FS	A12, A16	1428	*Citrus medica*	Intact protein (475aa)	+
Ichang papeda	YCC	A6, A15	1428	*Citrus ichangensis*	Intact protein (475aa)	+
Trifoliata orange	ZK	A8, A9	1428	*Poncirus trifoliata*	Intact protein (475aa)	+
Citrange	ZC	A10, A14	1428	*Citrus sinensis × Poncirus trifoliata*	Intact protein (475aa)	+
Citrumelo	ZY	A13, B1	1428/1433	*Poncirus trifoliata× Citrus paradisi*	Intact protein (475aa)/Truncated protein (174aa)	+
Star ruby grapefruit	XNB	A10, C2	1428/1433	*Citrus paradisi*	Intact protein (475aa)/Truncated protein (68aa)	+
Red marsh grapefruit	HMX	A10, C2	1428/1433	*Citrus paradisi*	Intact protein (475aa)/Truncated protein (68aa)	+
Marsh grapefruit	MX	A10, C2	1428/1433	*Citrus paradisi*	Intact protein (475aa)/Truncated protein (68aa)	+
Cocktail grapefruit	JW	A7, C2	1428/1433	*Citrus reticulata× Citrus maxima*	Intact protein (475aa)/Truncated protein (68aa)	+
Feicui pummelo	FC	B2, C2	1433/1427	*Citrus maxima*	Truncated protein (9aa)/Truncated protein (68aa)	−
Hiradobutun pummelo	HB	B2, C4	1433/1420	*Citrus maxima*	Truncated protein (9aa)/Truncated protein (68aa)	−
Chuhong pummelo	CH	B2, C3	1433/1427	*Citrus maxima*	Truncated protein (9aa)/Truncated protein (68aa)	−
Guanxi pummelo	GX	B2, C4	1433/1420	*Citrus maxima*	Truncated protein (9aa)/Truncated protein (68aa)	−
Shatian pummelo	ST	B4, C1	1433/1427		Truncated protein (174aa)/Truncated protein (68aa)	−
Kumquat	JG	C5, C6	1420/1420	*Citrus japonica*	Truncated protein (339aa)/Truncated protein (339aa)	−

“+” indicated accumulation of FRs; “-” indicated FRs were not detected.

### Allelic sequence variations of citrus 1,6RhaTs

To examine the correlation between the *1,6RhaT* genotype and the rutinoside accumulation phenotype, *1,6RhaT* was amplified from the DNA of 36 citrus accessions. A total of 28 different alleles were isolated with high similarity, and insertions, deletions and single nucleotide polymorphisms (SNPs) were also found. These alleles can be divided into three types, A, B and C, based on their sequences. Type A contains 18 alleles defined as A1 to A18, type B consists of B1 to B4, and C contains C1 to C6. Each accession investigated possessed two different alleles, with the exceptions of Bendizao tangerine and Guoqing No.1 Satsuma mandarin, which contained two identical alleles (Table 1).

All type A alleles consisted of 1428 bp with 9 ~ 41 SNP differences compared with *Cs1,6RhaT* (Supporting Table S3), which encodes a polypeptide of 475 amino acid (aa) residues and is speculated to produce complete functional 1,6RhaT proteins. Type A alleles can be amplified from some species, such as sweet orange and loose-skin mandarin, which accumulate FRs. Type B and C alleles ranged from 1420–1433 bp in length, shared 95.3%–96.0% nucleotide identity with the reference gene, and they generated premature proteins, which potentially do not exhibit 1,6RhaT functions (Supporting Table S4). Nonsense and frameshift mutations of the type B alleles were caused by the insertion of five base pairs, whereas base deletions at positions 185 or 984 led to frameshift mutations among the type C alleles. Additionally, more than 50 SNPs were observed for each member of the type B and C alleles. These alleles could be amplified from the genomes of pummelo, kumquat and hybrids such as grapefruit and citrons.

Among the type A alleles, A10 appeared most frequently and could be isolated from 18 accessions. For type B and type C alleles, the B2 allele showed the highest frequency during isolation.

**Figure 4:**
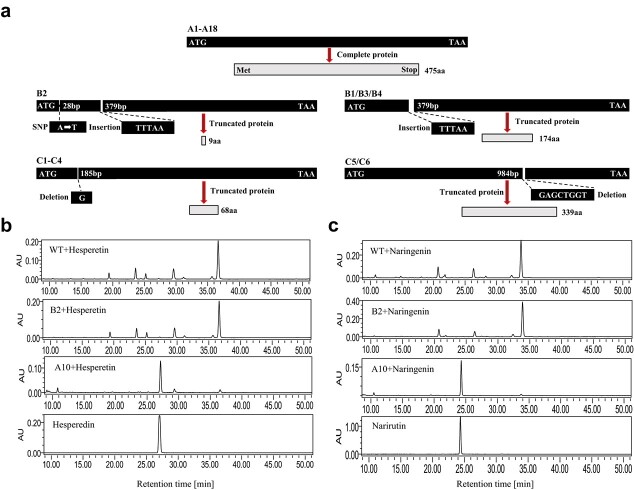
Schematic diagram of the structures of the 1,6RhaT alleles and functional characterization of 1,6RhaT alleles in tobacco BY2 cells. (a) Schematic diagram of the structures and deduced proteins of 1,6RhaT. (b) Functional characterization using hesperetin as the substrate. (c) Functional characterization using naringenin as the substrate. Met, methionine; aa, amino acid; WT, wild-type BY2 cells; B2, BY2 cells transgenic for the B2 allele; A10, BY2 cells transgenic for the A10 allele.

### Citrus *1,6RhaT* type a alleles encode proteins with 1,6RhaT function

As indicated above, premature stop codons in the type B and C alleles were hypothesized to produce truncated proteins without 1,6RhaT enzymatic function, which would lead to the unsuccessful accumulation of FRs. To validate this hypothesis, the catalytic activities of representative *1,6RhaT* alleles were analyzed in suspensions of transgenic BY2 tobacco calli. Wild-type BY2 and transgenic cell lines for the A10 and B2 alleles were given hesperetin or naringenin substrate for 48 h, and the resulting biotransformation products were extracted and identified by HPLC (Fig. 4b-c). New peaks were detected only in the transgenic cell lines for the A10 allele but not in the B2 allele and BY2 control. The products in the A10 allele cell lines were identified as hesperedin (hesperetin-7-*O*-rutinoside) and narirutin (naringenin-7-*O*-rutinoside) by comparison with standards. Thus, the substrate could be transformed into FRs in cells transformed with the A10 allele but this failed in cells with B2 and in the wild type, which indicated that only type A alleles have 1,6RhaT function.

### FR accumulation is correlated with the presence of type a alleles in citrus

The results from the *1,6RhaT* allele analysis with the FR contents among the 36 accessions suggested a significant association between allelic diversity and FR phenotypes (Supporting Table S5). FRs were detectable in accessions containing at least one type A allele, such as accessions belonging to sweet orange, loose-skin mandarin, trifoliate orange, ichang papeda and citrons. The undetectable FRs in pummelo and kumquat possessed type B and/or C but not type A alleles. Moreover, the contents of FRs in the accessions with two type A alleles (21.81 mg/g DW) were significantly higher than those with one type A allele (5.39 mg/g DW, *P* < 0.05). We hypothesized that the accumulation of FR was correlated with the presence of type A alleles in citrus.

**Figure 5:**
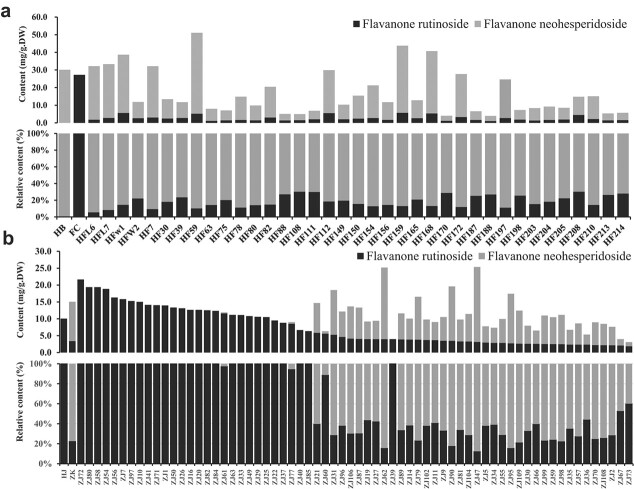
(a) FN and FR contents in the peel of the parent populations (HB pummelo and Fairchild tangelo) and the F1 hybrid populations. (b) FN and FR contents in the peel of the parent populations (red tangerine and trifoliata orange) and the F1 hybrid population. The peel include flavedo and albedo.

To confirm the hypothesis of the association between allelic diversity and FR phenotypes, populations of “HB pummelo”, “Fairchild tangelo” and their F_1_ hybrids were analyzed (Fig. 5a). The female parent HB pummelo contained B2 and C4 alleles with undetectable FRs in fruit peel, and the male parent “Fairchild tangelo” contained A5 and A10 with high levels of FRs in fruits (54.37 mg/g DW). Each F1 progeny obtained from the crossing carries a type B or C allele copy, and a type A allele copy, and thus it should accumulate FR (Supporting Fig. S1). HPLC analysis showed that all 37 F_1_ hybrids accumulated FR in the peel, which confirms our hypothesis.

### The proportion of FRs among flavanone disaccharides is affected by the genotypes of both *1,6RhaT* and *1,2RhaT* in the hybrids

The crossing of “HB pummelo” and “Fairchild tangelo” resulted in extremely significantly lower levels of FRs in the F1 hybrid population, which ranged from 1.05 to 5.71 mg/g DW with an average content of 2.54 mg/g DW in peel. The same result was also found in natural hybrids of trifoliate orange and grapefruit among the Group 2 accessions (Fig. 4). Therefore, a question raised in this study is what is the cause of the relatively low contents of FRs in these hybrids.

The abovementioned progenies contain functional *1,2RhaT* to produce FNs, which are a subgroup of flavanone disaccharides that compete with FRs converted from the same substrates of flavanone-7-*O-*glucosides (Supporting Fig. S2). Further determination of the contents of FNs (e.g. naringin and neohesperidin) showed that FRs were found at lower proportions, ranging from 5.53% to 30.20%, whereas FNs were found at proportions of 69.80%–94.47% (Fig. 5a), which implied that the synthesis of FNs might have negative influences on the accumulation of FRs.

To determine the above influences, F1 progenies resulting from another crossing of “red tangerine” and “trifoliata orange” were employed. Both the female and male parents in the crossing combination contained two type A *1,6RhaT* alleles (red tangerine: A4 and A10, trifoliate orange: A8 and A9) (Supplementary Fig. S3). However, *1,2RhaT* exists in the male parent “trifoliate orange” and 39 progenies but does not appear in the female parent “red tangerine” and 27 other progenies. High contents of FRs and undetectable levels of FNs were detected in the peel of “red tangerine”, and both FRs and FNs were detected in the peel of “trifoliate orange”. HPLC analysis showed that all 66 F_1_ hybrids accumulated FR in the peel, and both FRs and FNs were detectable in the abovementioned 39 progenies harboring *1,2RhaT* (Fig. 5b). However, the FR contents were lower than those of FNs in most of the 39 progenies (all except ZJ60, ZJ61, ZJ67, ZJ73 and ZJ77): FRs consisted of 12.43%–44.16% of all flavanone disaccharides and were found at levels of 2.17–5.58 mg/g DW with an average content of 3.29 mg/g DW, whereas FNs consisted of 55.84%–87.57% of all flavanone disaccharides and were found at contents of 2.98–22.21 mg/g DW with an average content of 8.40 mg/g DW (Supporting Table S6). These results showed that due to the involvement of FN biosynthesis, low FR contents were found in 39 of 66 F1 progenies from crossings of “red tangerine” and “trifoliata orange”, whereas high contents were detected in 27 other F1 progenies without FN biosynthesis. Therefore, it seems that the lower proportion of FRs among flavanone disaccharides was caused by the synthesis of FNs, which requires further in-depth research.

**Table 2:** Enzymatic kinetic parameters of Cs1,6RhaT and Cm1,2RhaT

	Substrate	*V*max (μM s^−1^ mg^−1^)	*K*m (μM)	*K*cat (min^−1^)	*K*cat/*K*m (s^−1^ mM^−1^)
	Naringenin-7-*O*-glucoside	22.2 ± 0.87	14.7 ± 0.21	7.02 ± 0.28	7.959
1,6RhaT	Hesperetin-7-*O*-glucoside	0.70 ± 0.05	12.9 ± 0.39	2.23 ± 0.17	2.884
	Naringenin-7-*O*-glucoside	34.4 ± 1.28	11.7 ± 2.11	10.6 ± 0.39	15.03
1,2RhaT	Hesperetin-7-*O*-glucoside	11.7 ± 0.83	25.5 ± 6.51	3.58 ± 0.26	2.346

### Enzymatic analyses of Cp1,6-RhaT (A10) and Cp1,2-RhaT

Furthermore, to clarify the influences of both 1,6RhaT and 1,2RhaT on FR production by considering substrate preference or competition, enzyme kinetic assays were performed *in vitro*. First, *1,6RhaT* and *1,2RhaT* were cloned from C. paradisi*,* which contains both genes. After the optimal temperature and pH for the reactions catalyzed by 1,2RhaT and 1,6RhaT were identified (Supporting Fig. S4), naringenin-7-*O*-glucoside (N7G) and hesperetin-7-*O*-glucoside (H7G) were used as substrates to determine the kinetic parameters because most citrus flavonoids are produced from these two substrates.

As shown in Table 2, the *K*m values of 1,6RhaT for N7G and H7G were 14.7 and 12.9 μM, respectively, whereas those of 1,2RhaT were 11.7 and 25.5 μM, respectively, which indicated that both enzymes exhibited strong binding affinity for these two compounds. The conversion efficiency (*K*cat) of 1,2RhaT for N7G and H7G was approximately 1.5-fold higher and approximately 1.6-fold higher than that of 1,6RhaT, respectively. Both 1,2RhaT and 1,6RhaT consumed N7G more efficiently than H7G. Analysis of the ratio of the catalytic efficiency to specificity (*K*cat/*K*m) for N7G showed that the ratio of 1,2RhaT was approximately 1.9-fold higher than that of 1,6RhaT. In contrast, the *K*cat/*K*m ratio of 1,2RhaT for H7G was lower due to the weaker affinity between 1,2RhaT and H7G.

These results showed that the efficiency of 1,2RhaT to form FNs was higher than that of 1,6RhaT to form FRs, which might partly explain the lower FR contents found in hybrids containing both *1,2RhaT* and *1,6RhaT*.

## Discussion

In our study, *1,6RhaT-like* genes were found in the genomes of relatively primitive species such as *A. buxifolia* and *C. medica* but were missing in model species such as loose-skin mandarin and sweet orange*.* Many reports have shown that plant flavanone disaccharides are related to resistance to environmental stress [24, 25]. For example, hesperidin accumulates in young leaves and fruits and plays an important role in defense against animal and microbial intrusion [26]. Naringin, hesperidin, and quercetin-3-*O*-rutinoside together with other active compounds can prevent Papilio machaon from laying eggs on young citrus leaves and can reduce the occurrence of citrus diseases and pests [27]. Identification of another *1,6RhaT* (*Cme1,6RhaT-like*) shows that multiple *1,6RhaT* genes work together to guarantee the synthesis of FRs in resisting the harsh environment of ancient times. As citrus began to be cultivated, *1,6RhaT-like* might have gradually became redundant due to the improvement of cultivation conditions and is now absent in modern germplasms.

**Figure 6:**
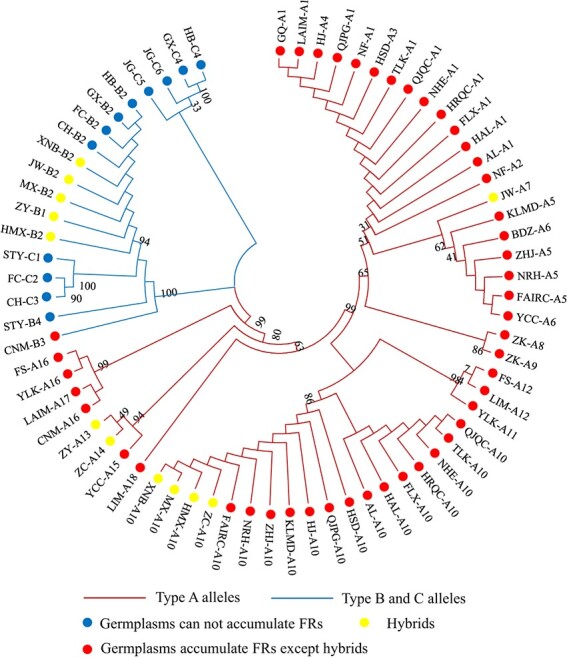
Phylogenic tree of 1,6RhaTs alleles in citrus accessions. Red lines represent type A alleles; blue lines represent type B and type C alleles; red circles represent accessions that accumulate FRs except hybrids; blue circles represent accessions that cannot accumulate FRs; green circles represent hybrids. Numbers indicate bootstrap replications. Abbreviations of the thirty-six citrus accessions are shown in Supplementary Table S1.

A phylogenetic tree was constructed based on the allelic variations among the 36 accessions, which not only successfully separated type A alleles from the other two types of alleles but also reflected complex genetic backgrounds among these accessions (Fig. 6). The results show that the type B and C alleles of hybrids such as grapefruits and “citrumelo” (ZY) were derived from pummelo, as these type B and C alleles clustered together. Type A alleles of grapefruits and citrange (ZC) were clustered with A10 of sweet orange, indicating that type A alleles of these accessions were derived from sweet orange. These results are consistent with the kinship of these species and accessions (grapefruit: sweet orange × pummelo; citrumelo: trifoliata orange × grapefruit; citrange: sweet orange × trifoliata orange). Interestingly, sweet orange results from a hybrid of pummelo and mandarin followed by a backcross with a mandarin [28] and therefore should have inherited the type B or C allele from the pummelo parent. Notably, we found that modern sweet orange does not contain type B or C alleles. We speculated that type B and C alleles existed in the initial hybrids of pummelo and mandarin; however, the hybrid not harboring type B and type C alleles that appeared during the backcross was selected for cultivation, which consequently led to the disappearance of type B and type C alleles in modern sweet orange.

The transcript levels of key genes are usually used to elucidate the accumulation of metabolites in various plants. However, the expression levels of *1,6RhaT*s showed marked differences among the 36 citrus accessions but were not well associated with the FR contents (Supporting Fig. S5). For example, the expression of *1,6RhaT* was detected in species such as pummelo and kumquat that do not accumulate FRs. This may be due to the simultaneous amplification of transcripts from nonfunctional *1,6RhaT*s (type B and C alleles) in these citrus species. High expression levels of *1,6RhaT* were detected in citrons that accumulate low levels of FRs, and previous research has shown that low enzyme levels of 1,6RhaT result in a low content of FRs in citron [19]; moreover, flavonoid-7-O-glucosides were not detected in citrus, indicating that the catalytic efficiency of 1,2RhaT and 1,6RhaT is very strong and that the supply of substrate is not saturated. Therefore, an insufficient substrate supply of 1,6RhaT may also be a cause of the low content of FR in citron.

The synthesis of citrus FNs and FRs involves two distinct but closely related competing subpathways during the rhamnosylation step that are catalyzed by *1,6RhaT* and *1,2RhaT*, respectively. Unlike *1,6RhaTs*, *1,2RhaT* has only one locus and it is directly related to the accumulation of FNs in citrus fruits, as demonstrated through an association analysis of 1,2RhaT with the FN accumulation phenotypes of 50 citrus accessions [20]. It is well known that FRs and FNs produce citrus fruits with nonbitter and bitter tastes, respectively, and these compounds have thus become important targets in citrus flavor-related breeding.

Our study found that the normal *1,6RhaT* (type A alleles) genotype is correlated with high levels of FRs in all investigated accessions, whereas accessions with early terminations of *1,6RhaT* resulting from frameshift mutations (type B and C alleles) had undetectable levels of FRs. Frameshift mutations are widespread in kiwifruit [29], Brassicaceae [30], pear [31] and apricot [32] crops and result in nonfunctional truncated proteins. However, exceptions were also found in grapefruit, trifoliata orange and its citrumelo hybrid harboring the type A allele and a lower FR content. These exceptions might be due to the influence of other genes related to the substrate supply, such as 7-*O*-glucosyltransferase, which catalyzes the formation of substrates of flavanone 7-*O*-glucosides, or to genes sharing the same substrates, such as *1,2RhaT*, which would account for the accumulation of FRs. The accumulation characteristics of flavanone glycosides in the two artificial hybrid populations also indicated that other factors might affect the accumulation of FRs.

Previous studies have shown that low enzyme levels of 1,6RhaT and high enzyme levels of 1,6RhaT were detected by corresponding antibodies in grapefruit and bitter orange, and different enzyme levels in germplasms containing both *1,6RhaT* and *1,2RhaT* may be one of the causes of germplasms with type A alleles but with low FR content [13, 19]. Moreover, we think that the catalytic efficiency of 1,2RhaT and 1,6RhaT is also a factor that affects the flavonoid content. Our results showed that the efficiency of 1,2RhaT to form FNs was higher than that of 1,6RhaT. This result is consistent with the flavonoid content in grapefruit, which might explain the lower FR contents from another perspective.

It is worth noting that kumquat, which contains functional *1,2RhaT* and nonfunctional type C *1,6RhaT* alleles, harbors another flavanone accumulation mode but no detectable FNs. This finding might be due to the loss or nonfunction of upstream genes that results in a lack of flavanone-7-*O*-glucoside supply [20].

Collectively, our results obtained in this study could partially explain the FR metabolic characteristics of various citrus species and can provide a better understanding of the biosynthesis and regulation of FRs, which is meaningful in theory and practice for improving citrus flavor and the functional components of flavonoid production. Using two distinct but closely related competing subpathways to reduce the bitter taste of citrus, we hope to obtain a certain degree of freedom to modulate the biosynthesis of flavanone disaccharides between FRs and FNs in the future.

## Acknowledgments

This work was supported by the National Key Research and Development Program of China (2018YFD1000204) and the National Natural Science Foundation of China (NSFC, Grant No. 32002010).

## Author contributions

W.Y.L. and G.L. performed the experiments and wrote the manuscript; M.L.T. established the hybrid populations; Z.Y.Y., M.Y.L., Y.H.M. and J.J.C. provided technical assistance and research guidance. J.X. designed the research and X.X.D. and J.X. revised the manuscript.

## Data availability

The data used to support the findings of this study are included within the article.

## Conflict of interest statement

The authors declare no competing interests.

## Supplementary data

Supplementary data is available at *Horticulture Research Journal* online.

## Supplementary Material

Web_Material_uhab017

## References

[1] Tripoli E , Guardia ML, Giammanco S et al. Citrus flavonoids: molecular structure, biological activity and nutritional properties: a review. Food Chem. 2007;104:466–79.

[2] Khan MK , Zill EH, Dangles O. A comprehensive review on flavanones, the major citrus polyphenols. J Food Compos Anal. 2014;33:85–104.

[3] Roohbakhsh A , Parhiz H, Soltani F et al. Neuropharmacological properties and pharmacokinetics of the citrus flavonoids hesperidin and hesperetin--a mini-review. Life Sci. 2014;113:1–6. 25109791 10.1016/j.lfs.2014.07.029

[4] Li C , Schluesener H. Health-promoting effects of the citrus flavanone hesperidin. Crit Rev Food Sci Nutr. 2017;57:613–31. 25675136 10.1080/10408398.2014.906382

[5] Tohge T , de Souza LP, Fernie AR. Current understanding of the pathways of flavonoid biosynthesis in model and crop plants. J Exp Bot. 2017;68:4013–28. 28922752 10.1093/jxb/erx177

[6] Wang L , Lee I-M, Zhang SM et al. Dietary intake of selected flavonols, flavones, and flavonoid-rich foods and risk of cancer in middle-aged and older women. Am J Clin Nutr. 2009;89:905–12. 19158208 10.3945/ajcn.2008.26913 PMC2667658

[7] Cheigh C-I , Chung E-Y, Chung M-S. Enhanced extraction of flavanones hesperidin and narirutin from Citrus unshiu peel using subcritical water. J Food Eng. 2012;110:472–7.

[8] Chen MC , Ye YY, Ji G et al. Hesperidin upregulates heme oxygenase-1 to attenuate hydrogen peroxide-induced cell damage in hepatic L02 cells. J Agric Food Chem. 2010;58:3330–5. 20170153 10.1021/jf904549s

[9] Constantin RP , Constantin RP, Bracht A et al. Molecular mechanisms of citrus flavanones on hepatic gluconeogenesis. Fitoterapia. 2014;92:148–62. 24239748 10.1016/j.fitote.2013.11.003

[10] Godos J , Castellano S, Ray S et al. Dietary polyphenol intake and depression: results from the Mediterranean healthy eating. Lifestyle and Aging (MEAL) Study *Molecules*. 2018;23:999. 10.3390/molecules23050999 PMC6102571 29695122

[11] Justin Thenmozhi A , William Raja TR, Manivasagam T et al. Hesperidin ameliorates cognitive dysfunction, oxidative stress and apoptosis against aluminium chloride induced rat model of Alzheimer's disease. Nutr Neurosci. 2017;20:360–8. 26878879 10.1080/1028415X.2016.1144846

[12] Berhow MA , Smolensky D. Developmental and substrate specificity of hesperetin-7-O-glucosyltransferase activity in citrus Limon tissues using high-performance liquid chromatographic analysis. Plant Sci. 1995;112:139–47.

[13] Frydman A , Weisshaus O, Bar-Peled M et al. Citrus fruit bitter flavors: isolation and functional characterization of the gene Cm1,2RhaT encoding a 1,2 rhamnosyltransferase, a key enzyme in the biosynthesis of the bitter flavonoids of citrus. Plant J. 2004;40:88–100. 15361143 10.1111/j.1365-313X.2004.02193.x

[14] Ohashi T , Hasegawa Y, Misaki R et al. Substrate preference of citrus naringenin rhamnosyltransferases and their application to flavonoid glycoside production in fission yeast. Appl Microbiol Biotechnol. 2016;100:687–96. 26433966 10.1007/s00253-015-6982-6

[15] Kawaii S , Tomono Y, Katase E et al. Quantitative study of fruit flavonoids in citrus hybrids of king (C. nobilis) and Mukaku Kishu (C. kinokuni). J Agric Food Chem. 2001;49:3982–6. 11513699 10.1021/jf0100292

[16] Kim HG , Kim G-S, Lee JH et al. Determination of the change of flavonoid components as the defence materials of Citrus unshiu Marc. Fruit peel against Penicillium digitatum by liquid chromatography coupled with tandem mass spectrometry. Food Chem. 2011;128:49–54. 25214328 10.1016/j.foodchem.2011.02.075

[17] Chen J , Zhang H, Pang Y et al. Comparative study of flavonoid production in lycopene-accumulated and blonde-flesh sweet oranges (Citrus sinensis) during fruit development. Food Chem. 2015;184:238–46. 25872450 10.1016/j.foodchem.2015.03.087

[18] Grilo FS , Di Stefano V, Lo Bianco R. Deficit irrigation and maturation stage influence quality and flavonoid composition of 'Valencia' orange fruit. J Sci Food Agric. 2017;97:1904–9. 27528197 10.1002/jsfa.7993

[19] Frydman A , Liberman R, Huhman DV et al. The molecular and enzymatic basis of bitter/non-bitter flavor of citrus fruit: evolution of branch-forming rhamnosyltransferases under domestication. Plant J. 2013;73:166–78. 22989156 10.1111/tpj.12030

[20] Chen J , Yuan Z, Zhang H et al. Cit1,2RhaT and two novel CitdGlcTs participate in flavor-related flavonoid metabolism during citrus fruit development. J Exp Bot. 2019;70:2759–71. 30840066 10.1093/jxb/erz081 PMC6506761

[21] Cheng Y-J , Guo W-W, Yi H-L et al. An efficient protocol for genomic DNA extraction fromCitrus species. Plant Mol Biol Report. 2003;21:177–8.

[22] Shaul O , Mironov V, Burssens S et al. Two Arabidopsis cyclin promoters mediate distinctive transcriptional oscillation in synchronized tobacco BY-2 cells. Proc Natl Acad Sci U S A. 1996;93:4868–72. 8643495 10.1073/pnas.93.10.4868 PMC39371

[23] Peng M , Shahzad R, Gul A et al. Differentially evolved glucosyltransferases determine natural variation of rice flavone accumulation and UV-tolerance. Nat Commun. 2017;8:1975. 29213047 10.1038/s41467-017-02168-x PMC5719032

[24] Devaiah SP , Owens DK, Sibhatu MB et al. Identification, recombinant expression, and biochemical analysis of putative secondary product Glucosyltransferases from Citrus paradisi. J Agric Food Chem. 2016;64:1957–69. 26888166 10.1021/acs.jafc.5b05430

[25] Peterson JJ , Dwyer JT, Beecher GR et al. Flavanones in oranges, tangerines (mandarins), tangors, and tangelos: a compilation and review of the data from the analytical literature. J Food Compos Anal. 2006;19:S66–73.

[26] del Río JA , Gomez P, Baidez AG et al. Changes in the levels of polymethoxyflavones and flavanones as part of the defense mechanism of Citrus sinensis (cv. Valencia late) fruits against Phytophthora citrophthora. J Agric Food Chem. 2004;52:1913–7. 15053528 10.1021/jf035038k

[27] Toh JY , Tan VM, Lim PC et al. Flavonoids from fruit and vegetables: a focus on cardiovascular risk factors. Curr Atheroscler Rep. 2013;15:368. 24091782 10.1007/s11883-013-0368-y

[28] Xu Q , Chen L-L, Ruan X et al. The draft genome of sweet orange (Citrus sinensis). Nat Genet. 2013;45:59–66. 23179022 10.1038/ng.2472

[29] Nieuwenhuizen NJ , Maddumage R, Tsang GK et al. Mapping, complementation, and targets of the cysteine protease actinidin in kiwifruit. Plant Physiol. 2012;158:376–88. 22039217 10.1104/pp.111.187989 PMC3252086

[30] Sun X , Pang H, Li M et al. Evolutionary pattern of the FAE1 gene in brassicaceae and its correlation with the erucic acid trait. PLoS One. 2013;8:e83535. 24358289 10.1371/journal.pone.0083535 PMC3865303

[31] Liu Y , Kong J, Li T et al. Isolation and characterization of an APETALA1-like gene from pear (Pyrus pyrifolia). Plant Mol Biol Report. 2013;31:1031–9.

[32] Zuriaga E , Romero C, Blanca JM et al. Resistance to plum pox virus (PPV) in apricot (Prunus armeniaca L.) is associated with down-regulation of two MATHd genes. BMC Plant Biol. 2018;18:25. 29374454 10.1186/s12870-018-1237-1 PMC5787289

