# Supplementary material for: Illustration of the variation in the content of flavanone rutinosides in various citrus germplasms from genetic and enzymatic perspectives
Source: Hortic Res. 2022 Jan 18;9:uhab017. doi: 10.1093/hr/uhab017 (PMC8788359; doi:10.1093/hr/uhab017)
Supplement: Web_Material_uhab017 [file web_material_uhab017.zip › Figure 1.pptx]

## Slide 1
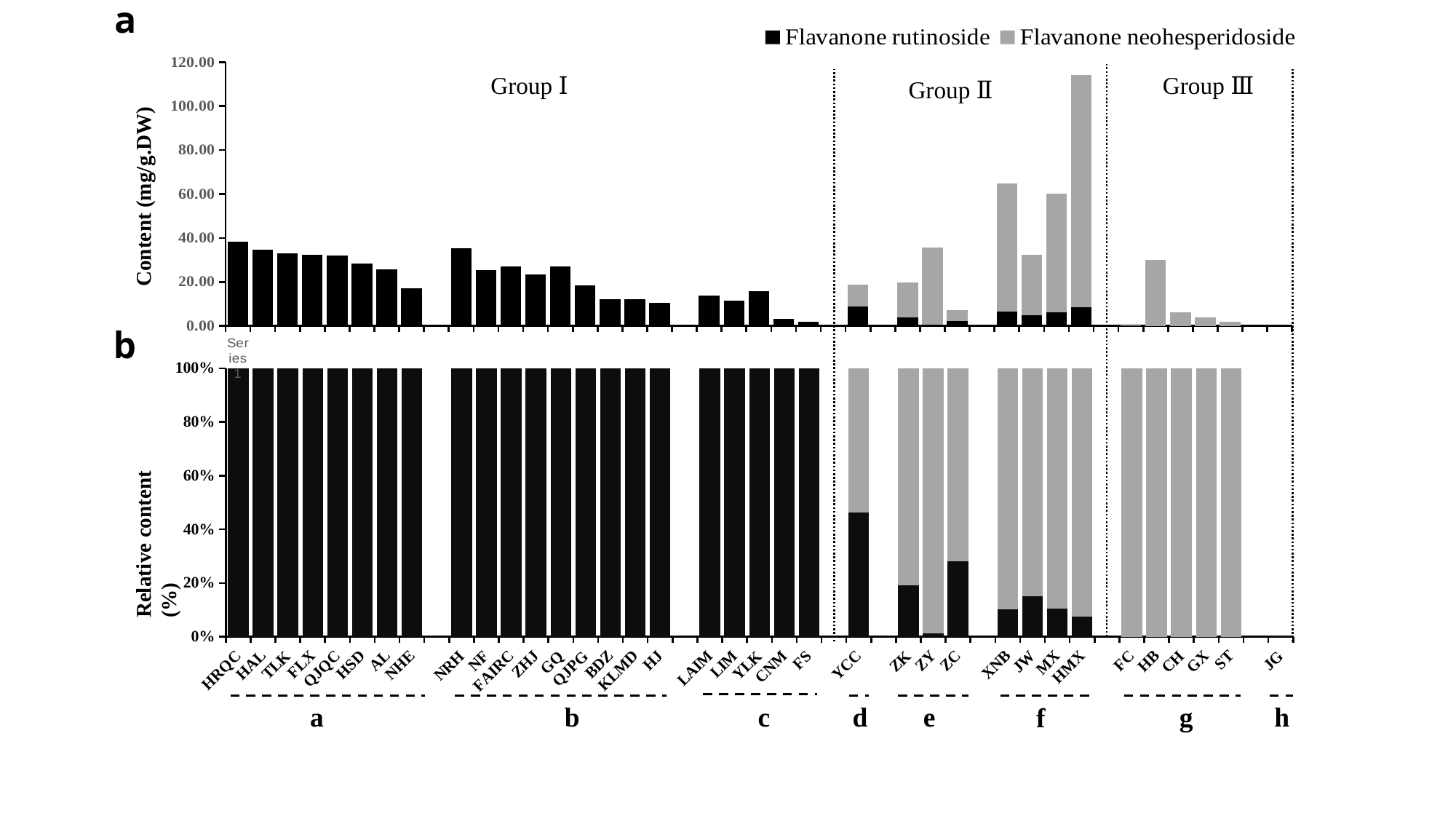

a
### Chart
| Category | Flavanone rutinoside | Flavanone neohesperidoside |
|---|---|---|
| | 38.1946723693217 | 0.0 |
| | 34.7787903792494 | 0.0 |
| | 33.1217809565292 | 0.0 |
| | 32.3159134362437 | 0.0 |
| | 32.1606222916081 | 0.0 |
| | 28.3696071208688 | 0.0 |
| | 25.7514836972868 | 0.0 |
| | 17.2354303465619 | 0.0 |
| | None | None |
| | 35.4236091583284 | 0.0 |
| | 25.4943868575974 | 0.0 |
| | 27.183351866347 | 0.0 |
| | 23.2589393585102 | 0.0 |
| | 27.0351351729617 | 0.0 |
| | 18.3022554181151 | 0.0 |
| | 12.2873054712258 | 0.0 |
| | 12.0458825420912 | 0.0 |
| | 10.4286636874096 | 0.0 |
| | None | None |
| | 13.7335360825764 | 0.0 |
| | 11.6245203813369 | 0.0 |
| | 15.9245948462025 | 0.0 |
| | 3.27816827813746 | 0.0 |
| | 1.91157886512922 | 0.0 |
| | None | None |
| | 8.72888901668933 | 10.109342733334 |
| | None | None |
| | 3.77494768948251 | 15.8727818708825 |
| | 0.415145278835584 | 35.2603809659973 |
| | 2.04841728377485 | 5.2603960375177 |
| | None | None |
| | 6.62431758720478 | 58.0395472921904 |
| | 4.90131927307119 | 27.6020825542967 |
| | 6.33201025769323 | 53.9041264563846 |
| | 8.66730299919138 | 105.592805758265 |
| | None | None |
| | 0.0 | 0.306568341197875 |
| | 0.0 | 30.0942652146786 |
| | 0.0 | 6.05796578530917 |
| | 0.0 | 3.73529711450059 |
| | 0.0 | 1.819868003357 |
| | None | None |
| | 0.0 | 0.0 |Group Ⅰ
Group Ⅲ
Group Ⅱ
Content (mg/g.DW)
### Chart
| Category | Flavanone rutinoside | Flavanone neohesperidoside |
|---|---|---|
| HRQC | 38.1946723693217 | 0.0 |
| HAL | 34.7787903792494 | 0.0 |
| TLK | 33.1217809565292 | 0.0 |
| FLX | 32.3159134362437 | 0.0 |
| QJQC | 32.1606222916081 | 0.0 |
| HSD | 28.3696071208688 | 0.0 |
| AL | 25.7514836972868 | 0.0 |
| NHE | 17.2354303465619 | 0.0 |
| | None | None |
| NRH | 35.4236091583284 | 0.0 |
| NF | 25.4943868575974 | 0.0 |
| FAIRC | 27.183351866347 | 0.0 |
| ZHJ | 23.2589393585102 | 0.0 |
| GQ | 27.0351351729617 | 0.0 |
| QJPG | 18.3022554181151 | 0.0 |
| BDZ | 12.2873054712258 | 0.0 |
| KLMD | 12.0458825420912 | 0.0 |
| HJ | 10.4286636874096 | 0.0 |
| | None | None |
| LAIM | 13.7335360825764 | 0.0 |
| LIM | 11.6245203813369 | 0.0 |
| YLK | 15.9245948462025 | 0.0 |
| CNM | 3.27816827813746 | 0.0 |
| FS | 1.91157886512922 | 0.0 |
| | None | None |
| YCC | 8.72888901668933 | 10.109342733334 |
| | None | None |
| ZK | 3.77494768948251 | 15.8727818708825 |
| ZY | 0.415145278835584 | 35.2603809659973 |
| ZC | 2.04841728377485 | 5.2603960375177 |
| | None | None |
| XNB | 6.62431758720478 | 58.0395472921904 |
| JW | 4.90131927307119 | 27.6020825542967 |
| MX | 6.33201025769323 | 53.9041264563846 |
| HMX | 8.66730299919138 | 105.592805758265 |
| | None | None |
| FC | 0.0 | 0.306568341197875 |
| HB | 0.0 | 30.0942652146786 |
| CH | 0.0 | 6.05796578530917 |
| GX | 0.0 | 3.73529711450059 |
| ST | 0.0 | 1.819868003357 |
| | None | None |
| JG | 0.0 | 0.0 |b
Relative content (%)
a
b
c
d
e
g
h
f
