# Supplementary material for: Illustration of the variation in the content of flavanone rutinosides in various citrus germplasms from genetic and enzymatic perspectives
Source: Hortic Res. 2022 Jan 18;9:uhab017. doi: 10.1093/hr/uhab017 (PMC8788359; doi:10.1093/hr/uhab017)
Supplement: Web_Material_uhab017 [file web_material_uhab017.zip › Figure 2.pptx]

## Slide 1
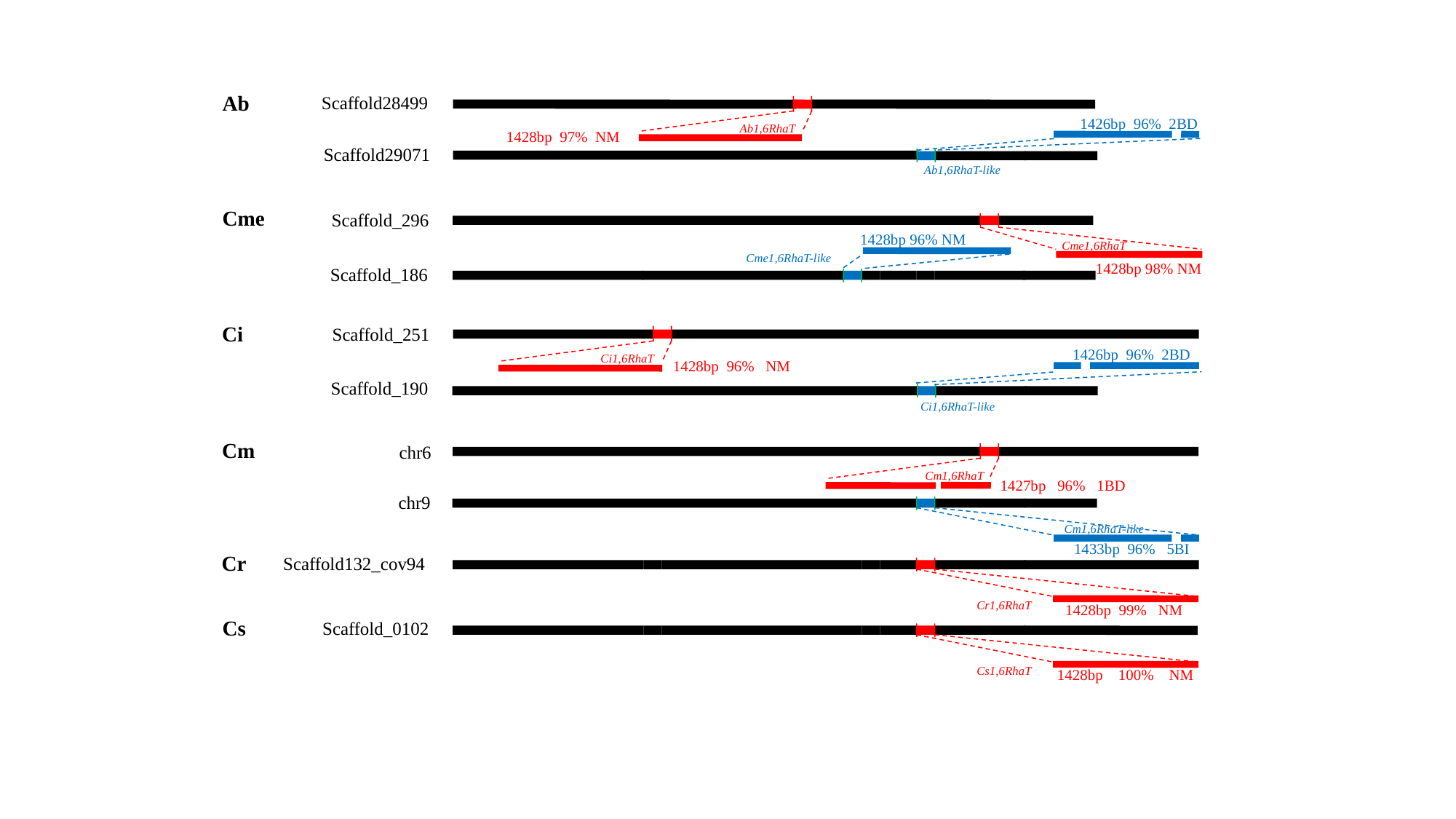

Ab
Scaffold28499
1426bp 96% 2BD
Ab1,6RhaT
1428bp 97% NM
Scaffold29071
Ab1,6RhaT-like
Cme
 Scaffold_296
1428bp 96% NM
Cme1,6RhaT
Cme1,6RhaT-like
 1428bp 98% NM
Scaffold_186
Ci
Scaffold_251
1426bp 96% 2BD
Ci1,6RhaT
1428bp 96% NM
Scaffold_190
Ci1,6RhaT-like
Cm
chr6
Cm1,6RhaT
1427bp 96% 1BD
chr9
Cm1,6RhaT-like
1433bp 96% 5BI
Cr
Scaffold132_cov94
Cr1,6RhaT
1428bp 99% NM
Cs
Scaffold_0102
Cs1,6RhaT
1428bp 100% NM
