# Supplementary material for: Illustration of the variation in the content of flavanone rutinosides in various citrus germplasms from genetic and enzymatic perspectives
Source: Hortic Res. 2022 Jan 18;9:uhab017. doi: 10.1093/hr/uhab017 (PMC8788359; doi:10.1093/hr/uhab017)
Supplement: Web_Material_uhab017 [file web_material_uhab017.zip › Figure 3.pptx]

## Slide 1
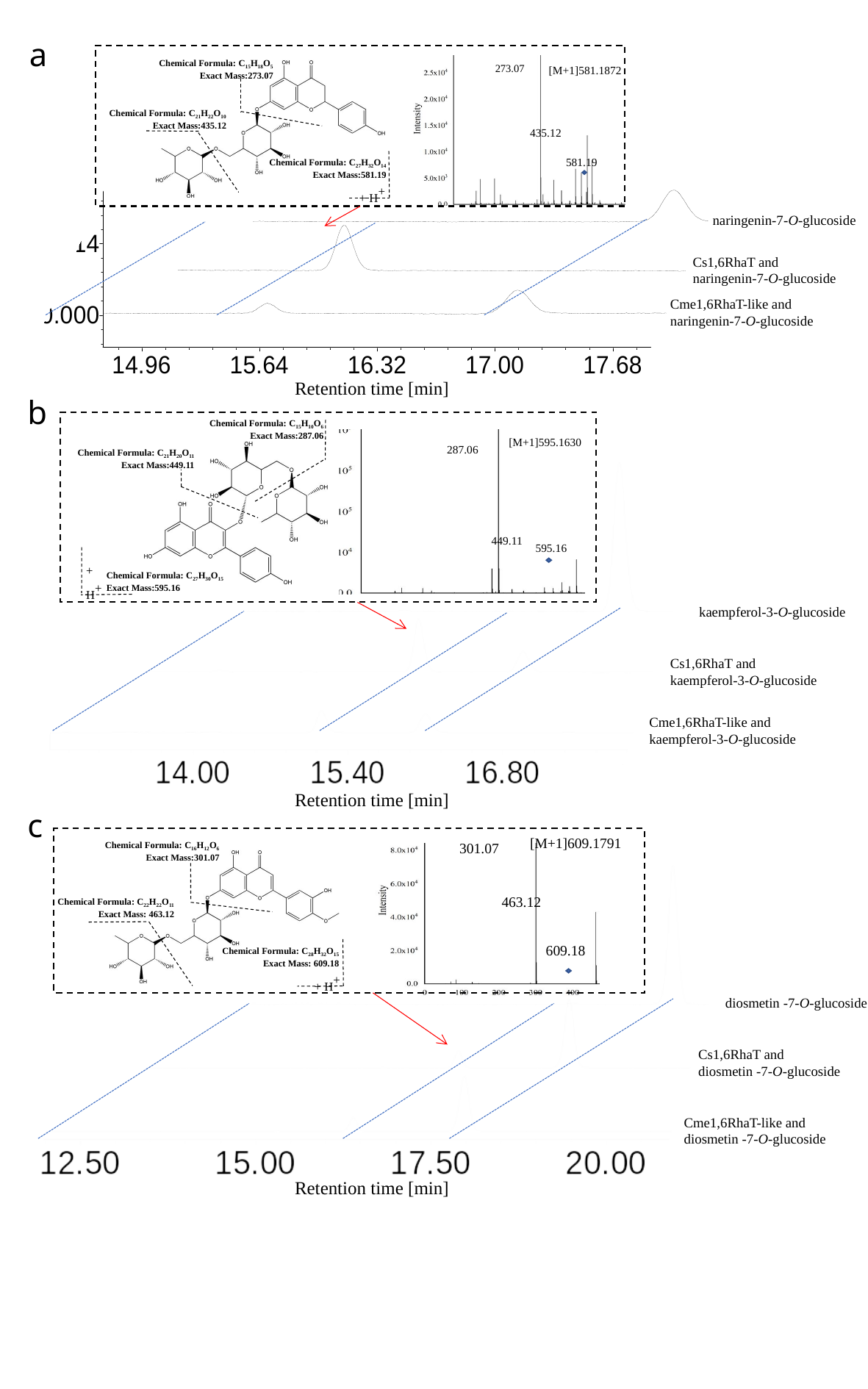

a
Chemical Formula: C15H18O5
Exact Mass:273.07
Chemical Formula: C21H22O10
Exact Mass:435.12
Chemical Formula: C27H32O14
Exact Mass:581.19
+ H+
273.07
[M+1]581.1872
435.12
581.19
naringenin-7-O-glucoside
Cs1,6RhaT and
naringenin-7-O-glucoside
Cme1,6RhaT-like and
naringenin-7-O-glucoside
Retention time [min]
b
Chemical Formula: C15H10O6
Exact Mass:287.06
[M+1]595.1630
287.06
449.11
595.16
Chemical Formula: C21H20O11
Exact Mass:449.11
+ H+
Chemical Formula: C27H30O15
Exact Mass:595.16
kaempferol-3-O-glucoside
Cs1,6RhaT and
kaempferol-3-O-glucoside
Cme1,6RhaT-like and
kaempferol-3-O-glucoside
Retention time [min]
c
[M+1]609.1791
301.07
463.12
609.18
Chemical Formula: C16H12O6
Exact Mass:301.07
Chemical Formula: C22H22O11
Exact Mass: 463.12
Chemical Formula: C28H32O15
Exact Mass: 609.18
+ H+
diosmetin -7-O-glucoside
Cs1,6RhaT and
diosmetin -7-O-glucoside
Cme1,6RhaT-like and
diosmetin -7-O-glucoside
Retention time [min]
