# Supplementary material for: Illustration of the variation in the content of flavanone rutinosides in various citrus germplasms from genetic and enzymatic perspectives
Source: Hortic Res. 2022 Jan 18;9:uhab017. doi: 10.1093/hr/uhab017 (PMC8788359; doi:10.1093/hr/uhab017)
Supplement: Web_Material_uhab017 [file web_material_uhab017.zip › Figure 4.pptx]

## Slide 1
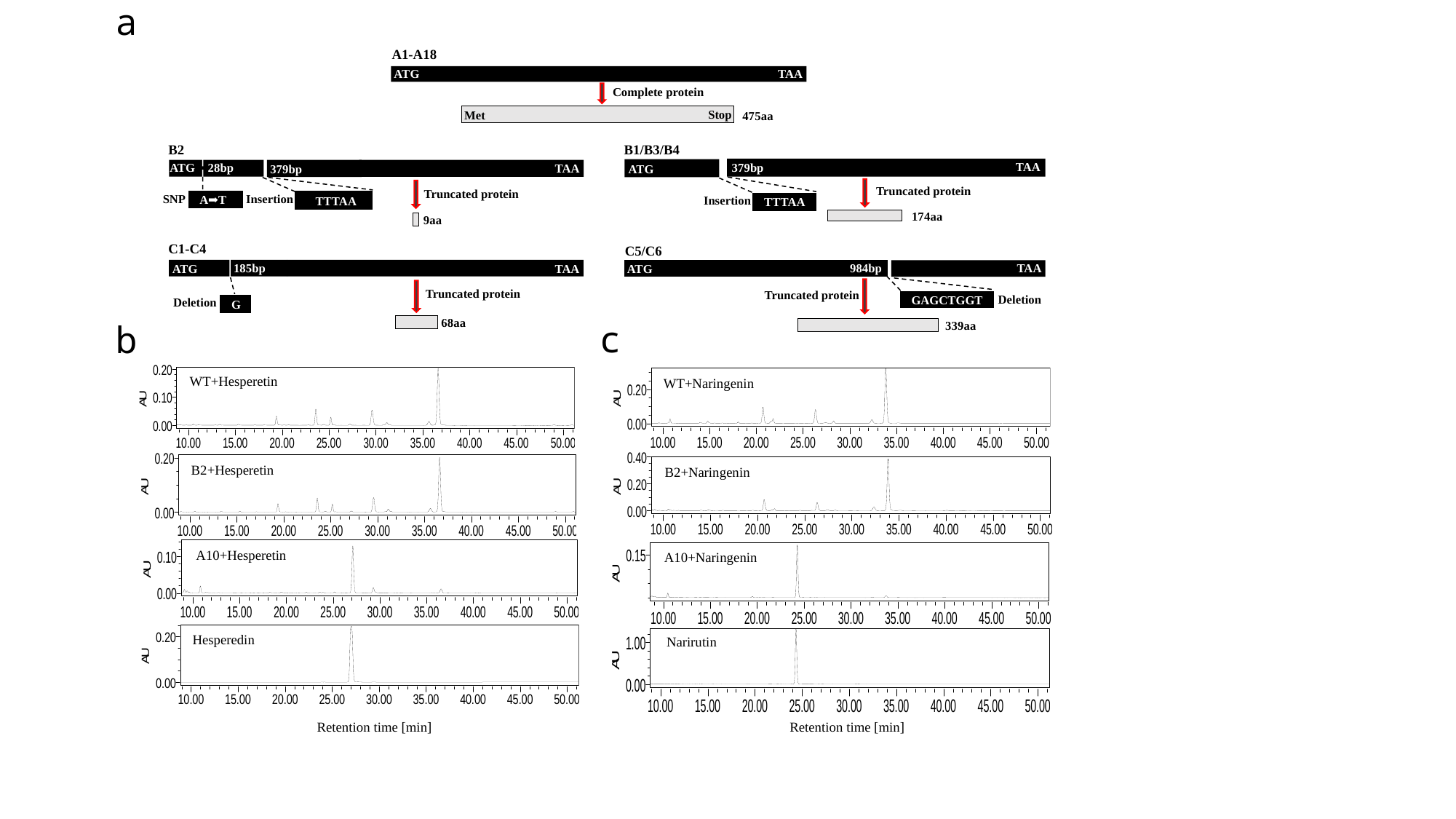

a
A1-A18
TAA
ATG
Complete protein
Stop
Met
475aa
B2
ATG
28bp
379bp
Truncated protein
SNP
Insertion
A➡T
 TTTAA
9aa
B1/B3/B4
379bp
ATG
Truncated protein
Insertion
TTTAA
TAA
TAA
TAA
174aa
C1-C4
185bp
ATG
ATG
Truncated protein
Deletion
G
68aa
C5/C6
TAA
984bp
TAA
ATG
Truncated protein
Deletion
GAGCTGGT
339aa
TAA
TAA
c
b
WT+Hesperetin
WT+Naringenin
B2+Hesperetin
B2+Naringenin
A10+Hesperetin
A10+Naringenin
Hesperedin
Narirutin
Retention time [min]
Retention time [min]
