# Supplementary material for: Illustration of the variation in the content of flavanone rutinosides in various citrus germplasms from genetic and enzymatic perspectives
Source: Hortic Res. 2022 Jan 18;9:uhab017. doi: 10.1093/hr/uhab017 (PMC8788359; doi:10.1093/hr/uhab017)
Supplement: Web_Material_uhab017 [file web_material_uhab017.zip › Figure 5.pptx]

## Slide 1
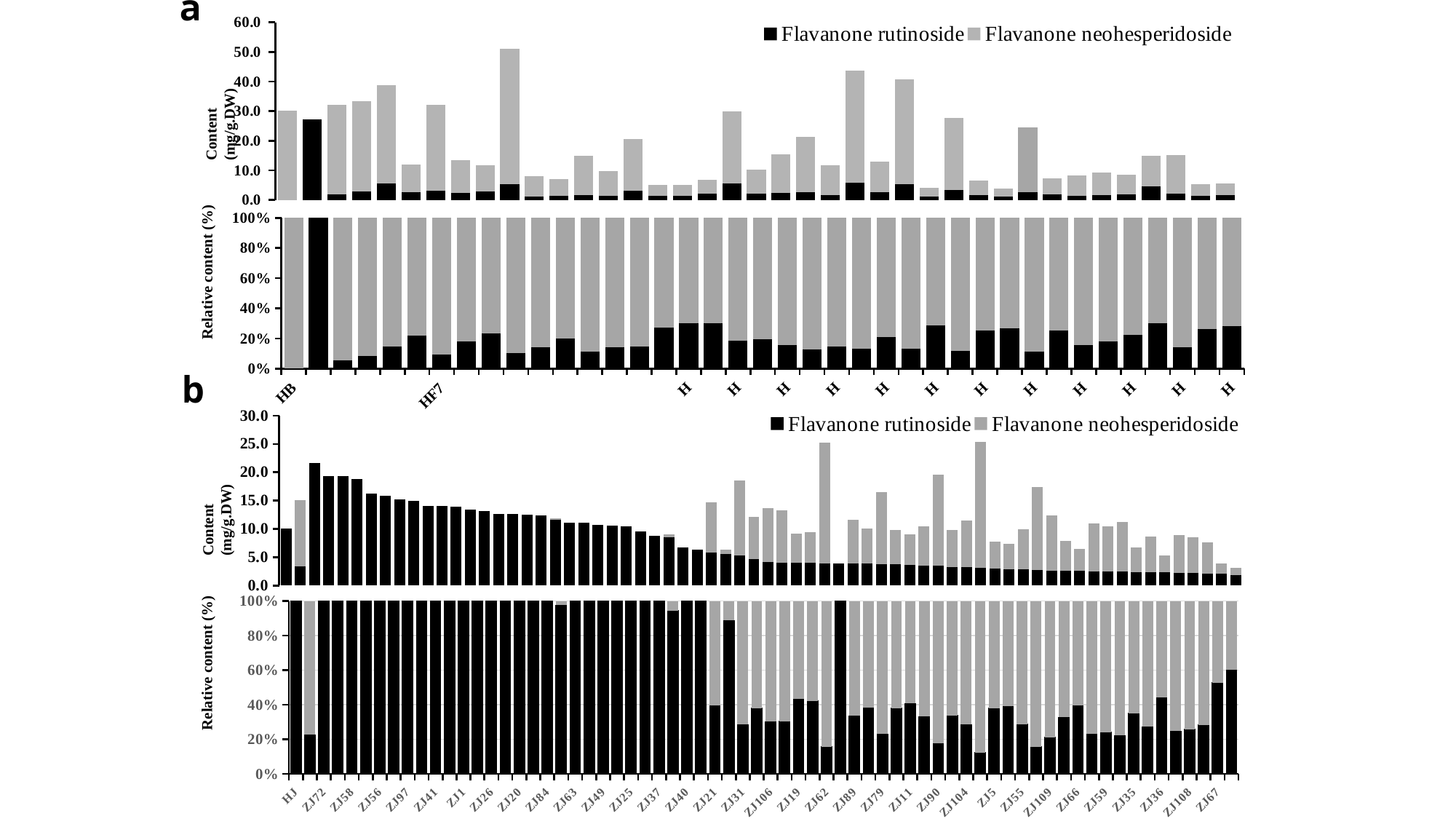

a
### Chart
| Category | | |
|---|---|---|
| HB | 0.0 | 30.094265214678586 |
| FC | 27.183351866347 | 0.0 |
| HFL6 | 1.7789131960972675 | 30.383823400284342 |
| HFL7 | 2.77175167223274 | 30.57594635404614 |
| HFw1 | 5.603152935630525 | 33.060934050888164 |
| HFW2 | 2.614368735592214 | 9.230536393369361 |
| HF7 | 3.0171023589746646 | 29.123523863679882 |
| HF30 | 2.4312228191624192 | 11.006251960839554 |
| HF39 | 2.7629003229322158 | 8.984185028218604 |
| HF59 | 5.221555359732238 | 45.86278410003007 |
| HF63 | 1.1345095827780487 | 6.808667451607633 |
| HF75 | 1.4221224847896983 | 5.624262440761599 |
| HF78 | 1.6554656714839389 | 13.194427783522519 |
| HF80 | 1.3882442811648474 | 8.440863695350496 |
| HF82 | 3.0217738890161776 | 17.4213705755175 |
| HF88 | 1.372552563675014 | 3.6910096649156188 |
| HF108 | 1.4953774919902063 | 3.4555167374662905 |
| HF111 | 2.0527669772774018 | 4.805573295055074 |
| HF112 | 5.5346196393854 | 24.39190210622534 |
| HF149 | 2.0114328154646532 | 8.300558255880778 |
| HF150 | 2.416993560797889 | 13.037105682788939 |
| HF154 | 2.706958696920891 | 18.51217019123825 |
| HF156 | 1.6966863541395067 | 10.065793181072854 |
| HF159 | 5.713231192509156 | 38.043856081875305 |
| HF165 | 2.6634402010725644 | 10.179453606288346 |
| HF168 | 5.344986602478841 | 35.349288975295345 |
| HF170 | 1.1633797167170472 | 2.880354631972439 |
| HF172 | 3.2974902714264656 | 24.396625454893268 |
| HF187 | 1.6688696804200382 | 4.918174079157841 |
| HF188 | 1.0495374574593495 | 2.8597673418429452 |
| HF197 | 2.72576886039525 | 21.86501664603075 |
| HF198 | 1.8550273782905793 | 5.426180973857548 |
| HF203 | 1.2933566209523149 | 7.0652421881735075 |
| HF204 | 1.6793452788133492 | 7.555527754415311 |
| HF205 | 1.8925285595839132 | 6.61099371293172 |
| HF208 | 4.476840884138496 | 10.35629917578924 |
| HF210 | 2.168807721071427 | 12.912516600265604 |
| HF213 | 1.4131905114712406 | 3.936239539200793 |
| HF214 | 1.5862294916913047 | 4.068063449912528 |Content (mg/g.DW)
### Chart
| Category | | |
|---|---|---|
| HB | 0.0 | 30.094265214678586 |
| FC | 27.183351866347 | 0.0 |
| HFL6 | 1.7789131960972675 | 30.383823400284342 |
| HFL7 | 2.77175167223274 | 30.57594635404614 |
| HFw1 | 5.603152935630525 | 33.060934050888164 |
| HFW2 | 2.614368735592214 | 9.230536393369361 |
| HF7 | 3.0171023589746646 | 29.123523863679882 |
| HF30 | 2.4312228191624192 | 11.006251960839554 |
| HF39 | 2.7629003229322158 | 8.984185028218604 |
| HF59 | 5.221555359732238 | 45.86278410003007 |
| HF63 | 1.1345095827780487 | 6.808667451607633 |
| HF75 | 1.4221224847896983 | 5.624262440761599 |
| HF78 | 1.6554656714839389 | 13.194427783522519 |
| HF80 | 1.3882442811648474 | 8.440863695350496 |
| HF82 | 3.0217738890161776 | 17.4213705755175 |
| HF88 | 1.372552563675014 | 3.6910096649156188 |
| HF108 | 1.4953774919902063 | 3.4555167374662905 |
| HF111 | 2.0527669772774018 | 4.805573295055074 |
| HF112 | 5.5346196393854 | 24.39190210622534 |
| HF149 | 2.0114328154646532 | 8.300558255880778 |
| HF150 | 2.416993560797889 | 13.037105682788939 |
| HF154 | 2.706958696920891 | 18.51217019123825 |
| HF156 | 1.6966863541395067 | 10.065793181072854 |
| HF159 | 5.713231192509156 | 38.043856081875305 |
| HF165 | 2.6634402010725644 | 10.179453606288346 |
| HF168 | 5.344986602478841 | 35.349288975295345 |
| HF170 | 1.1633797167170472 | 2.880354631972439 |
| HF172 | 3.2974902714264656 | 24.396625454893268 |
| HF187 | 1.6688696804200382 | 4.918174079157841 |
| HF188 | 1.0495374574593495 | 2.8597673418429452 |
| HF197 | 2.72576886039525 | 21.86501664603075 |
| HF198 | 1.8550273782905793 | 5.426180973857548 |
| HF203 | 1.2933566209523149 | 7.0652421881735075 |
| HF204 | 1.6793452788133492 | 7.555527754415311 |
| HF205 | 1.8925285595839132 | 6.61099371293172 |
| HF208 | 4.476840884138496 | 10.35629917578924 |
| HF210 | 2.168807721071427 | 12.912516600265604 |
| HF213 | 1.4131905114712406 | 3.936239539200793 |
| HF214 | 1.5862294916913047 | 4.068063449912528 |Relative content (%)
b
### Chart
| Category | Flavanone rutinoside | Flavanone neohesperidoside |
|---|---|---|
| HJ | 10.0647863527679 | 0.0 |
| ZK | 3.3989128453666 | 11.6533278717084 |
| ZJ72 | 21.6375751431592 | 0.0 |
| ZJ80 | 19.3764665927937 | 0.0 |
| ZJ58 | 19.3759483450031 | 0.0 |
| ZJ54 | 18.8648198505171 | 0.0 |
| ZJ56 | 16.3060898144489 | 0.0 |
| ZJ7 | 15.8314091684743 | 0.0 |
| ZJ97 | 15.2753414687626 | 0.0 |
| ZJ10 | 15.0147662739474 | 0.0 |
| ZJ41 | 14.1175322818983 | 0.0 |
| ZJ71 | 14.0400372534781 | 0.0 |
| ZJ1 | 13.9555568742291 | 0.0 |
| ZJ50 | 13.3585591322486 | 0.0 |
| ZJ26 | 13.1095400336695 | 0.0 |
| ZJ16 | 12.6439510875215 | 0.0 |
| ZJ20 | 12.6421219491171 | 0.0 |
| ZJ82 | 12.5301223101979 | 0.0 |
| ZJ84 | 12.3629923439586 | 0.0 |
| ZJ61 | 11.6130861499864 | 0.294864652516078 |
| ZJ63 | 11.156128613983 | 0.0 |
| ZJ33 | 11.1193663392754 | 0.0 |
| ZJ49 | 10.7750886430486 | 0.0 |
| ZJ29 | 10.6121452308484 | 0.0 |
| ZJ25 | 10.4975192417696 | 0.0 |
| ZJ22 | 9.5181599961845 | 0.0 |
| ZJ37 | 8.80978941678173 | 0.0 |
| ZJ77 | 8.53337892526279 | 0.48973320870928 |
| ZJ40 | 6.6767143672022 | 0.0 |
| ZJ85 | 6.34918626288652 | 0.0 |
| ZJ21 | 5.85062932264024 | 8.83889547022853 |
| ZJ60 | 5.60382188504081 | 0.70777688565466 |
| ZJ31 | 5.28977052390166 | 13.2120283008831 |
| ZJ96 | 4.62761238304215 | 7.53377466607493 |
| ZJ106 | 4.12958001314829 | 9.54782165695436 |
| ZJ87 | 4.04283353672422 | 9.27295219266425 |
| ZJ19 | 3.99593390130532 | 5.18426091423833 |
| ZJ27 | 3.97791200364784 | 5.42836140645045 |
| ZJ62 | 3.9550165734342 | 21.2371304934681 |
| ZJ39 | 3.95035513437418 | 0.0 |
| ZJ89 | 3.89948163538561 | 7.72634237213615 |
| ZJ14 | 3.84706567574905 | 6.19646769200633 |
| ZJ79 | 3.83325512393356 | 12.7018193906531 |
| ZJ102 | 3.71547856896352 | 6.07436207662657 |
| ZJ11 | 3.69017358630759 | 5.35636674033731 |
| ZJ9 | 3.48413403675407 | 7.0301857164501 |
| ZJ90 | 3.48080755783109 | 16.1099182264 |
| ZJ81 | 3.30077456091259 | 6.47140694632767 |
| ZJ104 | 3.27223325134212 | 8.16167795456028 |
| ZJ47 | 3.15170891770873 | 22.2084484686462 |
| ZJ5 | 2.94723736444958 | 4.82446866418636 |
| ZJ34 | 2.86884743269865 | 4.4888400281742 |
| ZJ55 | 2.86714816292461 | 7.10434793333867 |
| ZJ95 | 2.73757121978593 | 14.6718744478203 |
| ZJ109 | 2.64112253071542 | 9.77540199106308 |
| ZJ30 | 2.60608461042793 | 5.34605279279707 |
| ZJ66 | 2.59002129923697 | 3.92799777614162 |
| ZJ99 | 2.54171884246758 | 8.41159483511751 |
| ZJ59 | 2.51554586043615 | 7.93947664280409 |
| ZJ98 | 2.4947055398194 | 8.67611996355949 |
| ZJ35 | 2.36369181557264 | 4.37423726223881 |
| ZJ57 | 2.36085529950965 | 6.27298315812496 |
| ZJ36 | 2.35805420966714 | 2.98182659986989 |
| ZJ70 | 2.22591414871814 | 6.740889735023 |
| ZJ108 | 2.18681192034468 | 6.29561449967327 |
| ZJ2 | 2.16696799326183 | 5.45764646802262 |
| ZJ67 | 2.09110533916406 | 1.87238535315553 |
| ZJ73 | 1.86165746872154 | 1.23517998748286 |Content (mg/g.DW)
Relative content (%)
### Chart
| Category | Flavanone rutinoside | Flavanone neohesperidoside |
|---|---|---|
| HJ | 10.0647863527679 | 0.0 |
| ZK | 3.3989128453666 | 11.6533278717084 |
| ZJ72 | 21.6375751431592 | 0.0 |
| ZJ80 | 19.3764665927937 | 0.0 |
| ZJ58 | 19.3759483450031 | 0.0 |
| ZJ54 | 18.8648198505171 | 0.0 |
| ZJ56 | 16.3060898144489 | 0.0 |
| ZJ7 | 15.8314091684743 | 0.0 |
| ZJ97 | 15.2753414687626 | 0.0 |
| ZJ10 | 15.0147662739474 | 0.0 |
| ZJ41 | 14.1175322818983 | 0.0 |
| ZJ71 | 14.0400372534781 | 0.0 |
| ZJ1 | 13.9555568742291 | 0.0 |
| ZJ50 | 13.3585591322486 | 0.0 |
| ZJ26 | 13.1095400336695 | 0.0 |
| ZJ16 | 12.6439510875215 | 0.0 |
| ZJ20 | 12.6421219491171 | 0.0 |
| ZJ82 | 12.5301223101979 | 0.0 |
| ZJ84 | 12.3629923439586 | 0.0 |
| ZJ61 | 11.6130861499864 | 0.294864652516078 |
| ZJ63 | 11.156128613983 | 0.0 |
| ZJ33 | 11.1193663392754 | 0.0 |
| ZJ49 | 10.7750886430486 | 0.0 |
| ZJ29 | 10.6121452308484 | 0.0 |
| ZJ25 | 10.4975192417696 | 0.0 |
| ZJ22 | 9.5181599961845 | 0.0 |
| ZJ37 | 8.80978941678173 | 0.0 |
| ZJ77 | 8.53337892526279 | 0.48973320870928 |
| ZJ40 | 6.6767143672022 | 0.0 |
| ZJ85 | 6.34918626288652 | 0.0 |
| ZJ21 | 5.85062932264024 | 8.83889547022853 |
| ZJ60 | 5.60382188504081 | 0.70777688565466 |
| ZJ31 | 5.28977052390166 | 13.2120283008831 |
| ZJ96 | 4.62761238304215 | 7.53377466607493 |
| ZJ106 | 4.12958001314829 | 9.54782165695436 |
| ZJ87 | 4.04283353672422 | 9.27295219266425 |
| ZJ19 | 3.99593390130532 | 5.18426091423833 |
| ZJ27 | 3.97791200364784 | 5.42836140645045 |
| ZJ62 | 3.9550165734342 | 21.2371304934681 |
| ZJ39 | 3.95035513437418 | 0.0 |
| ZJ89 | 3.89948163538561 | 7.72634237213615 |
| ZJ14 | 3.84706567574905 | 6.19646769200633 |
| ZJ79 | 3.83325512393356 | 12.7018193906531 |
| ZJ102 | 3.71547856896352 | 6.07436207662657 |
| ZJ11 | 3.69017358630759 | 5.35636674033731 |
| ZJ9 | 3.48413403675407 | 7.0301857164501 |
| ZJ90 | 3.48080755783109 | 16.1099182264 |
| ZJ81 | 3.30077456091259 | 6.47140694632767 |
| ZJ104 | 3.27223325134212 | 8.16167795456028 |
| ZJ47 | 3.15170891770873 | 22.2084484686462 |
| ZJ5 | 2.94723736444958 | 4.82446866418636 |
| ZJ34 | 2.86884743269865 | 4.4888400281742 |
| ZJ55 | 2.86714816292461 | 7.10434793333867 |
| ZJ95 | 2.73757121978593 | 14.6718744478203 |
| ZJ109 | 2.64112253071542 | 9.77540199106308 |
| ZJ30 | 2.60608461042793 | 5.34605279279707 |
| ZJ66 | 2.59002129923697 | 3.92799777614162 |
| ZJ99 | 2.54171884246758 | 8.41159483511751 |
| ZJ59 | 2.51554586043615 | 7.93947664280409 |
| ZJ98 | 2.4947055398194 | 8.67611996355949 |
| ZJ35 | 2.36369181557264 | 4.37423726223881 |
| ZJ57 | 2.36085529950965 | 6.27298315812496 |
| ZJ36 | 2.35805420966714 | 2.98182659986989 |
| ZJ70 | 2.22591414871814 | 6.740889735023 |
| ZJ108 | 2.18681192034468 | 6.29561449967327 |
| ZJ2 | 2.16696799326183 | 5.45764646802262 |
| ZJ67 | 2.09110533916406 | 1.87238535315553 |
| ZJ73 | 1.86165746872154 | 1.23517998748286 |
