# Supplementary material for: Illustration of the variation in the content of flavanone rutinosides in various citrus germplasms from genetic and enzymatic perspectives
Source: Hortic Res. 2022 Jan 18;9:uhab017. doi: 10.1093/hr/uhab017 (PMC8788359; doi:10.1093/hr/uhab017)
Supplement: Web_Material_uhab017 [file web_material_uhab017.zip › Figure 6.pptx]

## Slide 1
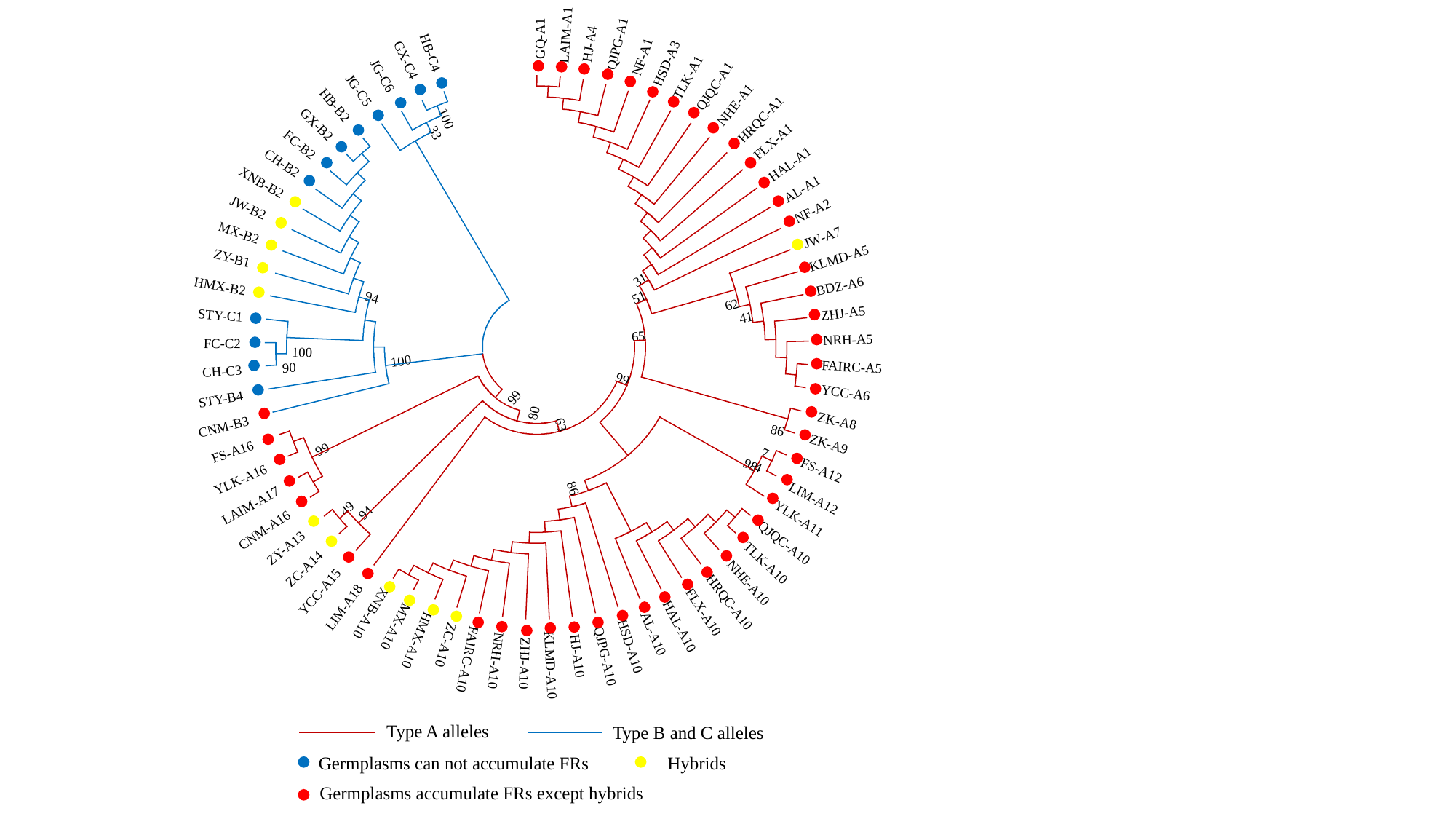

LAIM-A1
GQ-A1
QJPG-A1
HJ-A4
NF-A1
HSD-A3
TLK-A1
QJQC-A1
NHE-A1
HRQC-A1
FLX-A1
HAL-A1
AL-A1
NF-A2
JW-A7
KLMD-A5
31
BDZ-A6
51
62
ZHJ-A5
41
65
NRH-A5
FAIRC-A5
YCC-A6
ZK-A8
86
ZK-A9
74
98
FS-A12
LIM-A12
YLK-A11
QJQC-A10
TLK-A10
NHE-A10
HRQC-A10
FLX-A10
HAL-A10
AL-A10
HSD-A10
HJ-A10
QJPG-A10
FAIRC-A10
NRH-A10
ZHJ-A10
KLMD-A10
HB-C4
GX-C4
JG-C6
JG-C5
HB-B2
100
GX-B2
FC-B2
CH-B2
XNB-B2
JW-B2
MX-B2
ZY-B1
HMX-B2
94
STY-C1
FC-C2
100
100
90
CH-C3
99
99
STY-B4
80
63
CNM-B3
99
FS-A16
YLK-A16
86
LAIM-A17
49
94
CNM-A16
ZY-A13
ZC-A14
YCC-A15
LIM-A18
XNB-A10
MX-A10
HMX-A10
ZC-A10
33
Type A alleles
Type B and C alleles
Hybrids
Germplasms can not accumulate FRs
Germplasms accumulate FRs except hybrids
