# Supplementary material for: Illustration of the variation in the content of flavanone rutinosides in various citrus germplasms from genetic and enzymatic perspectives
Source: Hortic Res. 2022 Jan 18;9:uhab017. doi: 10.1093/hr/uhab017 (PMC8788359; doi:10.1093/hr/uhab017)
Supplement: Web_Material_uhab017 [file web_material_uhab017.zip › Supplementary Figure S1-S5.docx]

Supplementary figures for

**Illustration of the variation in the content of flavanone rutinosides in various citrus germplasms from genetic and enzymatic perspectives**

Wenyun Li^1,2#^, Gu Li^1#^, Ziyu Yuan^1^, Mingyue Li^1^, Xiuxin Deng^1^, Meilian Tan^3^, Yuhua Ma^2^, Jiajing Chen^1*^, Juan Xu^1*^

Institution addresses:

1 Key Laboratory of Horticultural Plant Biology (Ministry of Education), College of Horticulture and Forestry, Huazhong Agricultural University, Wuhan 430070, China.

2 Guizhou Fruit Institute, Guizhou Academy of Agricultural Sciences, Guiyang 550006, China.

3 The Oil Crops Research Institute of the Chinese Academy of Agricultural Sciences, Wuhan 430062, China

#These authors contribute equally to the manuscript.

* **Corresponding authors:**

Jiajing Chen, E-mail: jiajingchen@webmail.hzau.edu.cn.

**Juan Xu** Tel: +86-027-87286965, Email: [xujuan@mail.hzau.edu.cn](mailto:xujuan@mail.hzau.edu.cn)

ORCID: 0000-0003-4623-9343


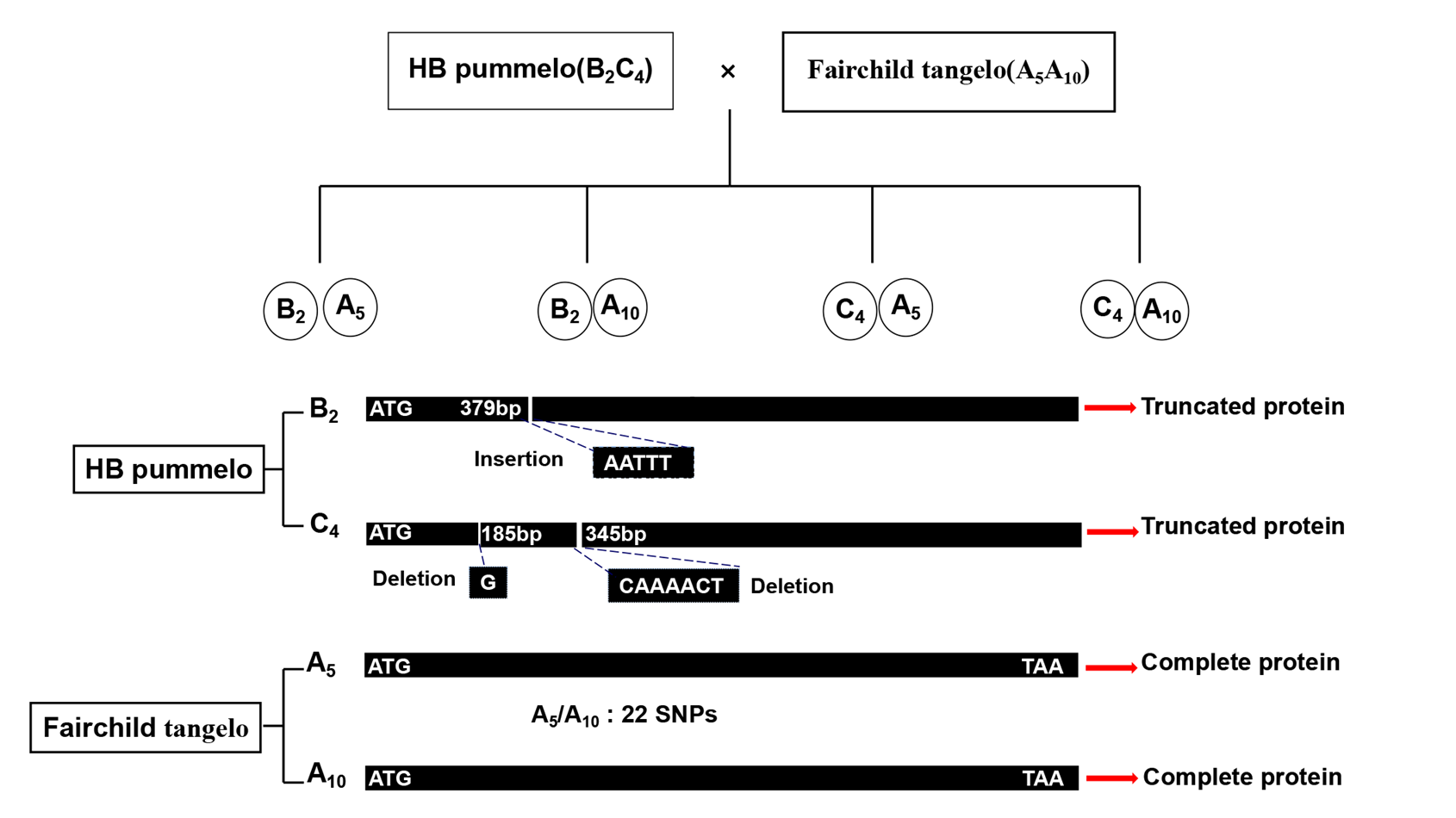


**Fig. S1** **Sequence analysis of 1,6RhaT in HB pummelo and Fairchild tangelo and genotype prediction of F1 progenies.** The nucleotide sequences were in black; Dotted line indicated insertion/deletion position on the nucleotide sequence. ATG, Initiation codon; TAA, Termination codon; Met, methionine.


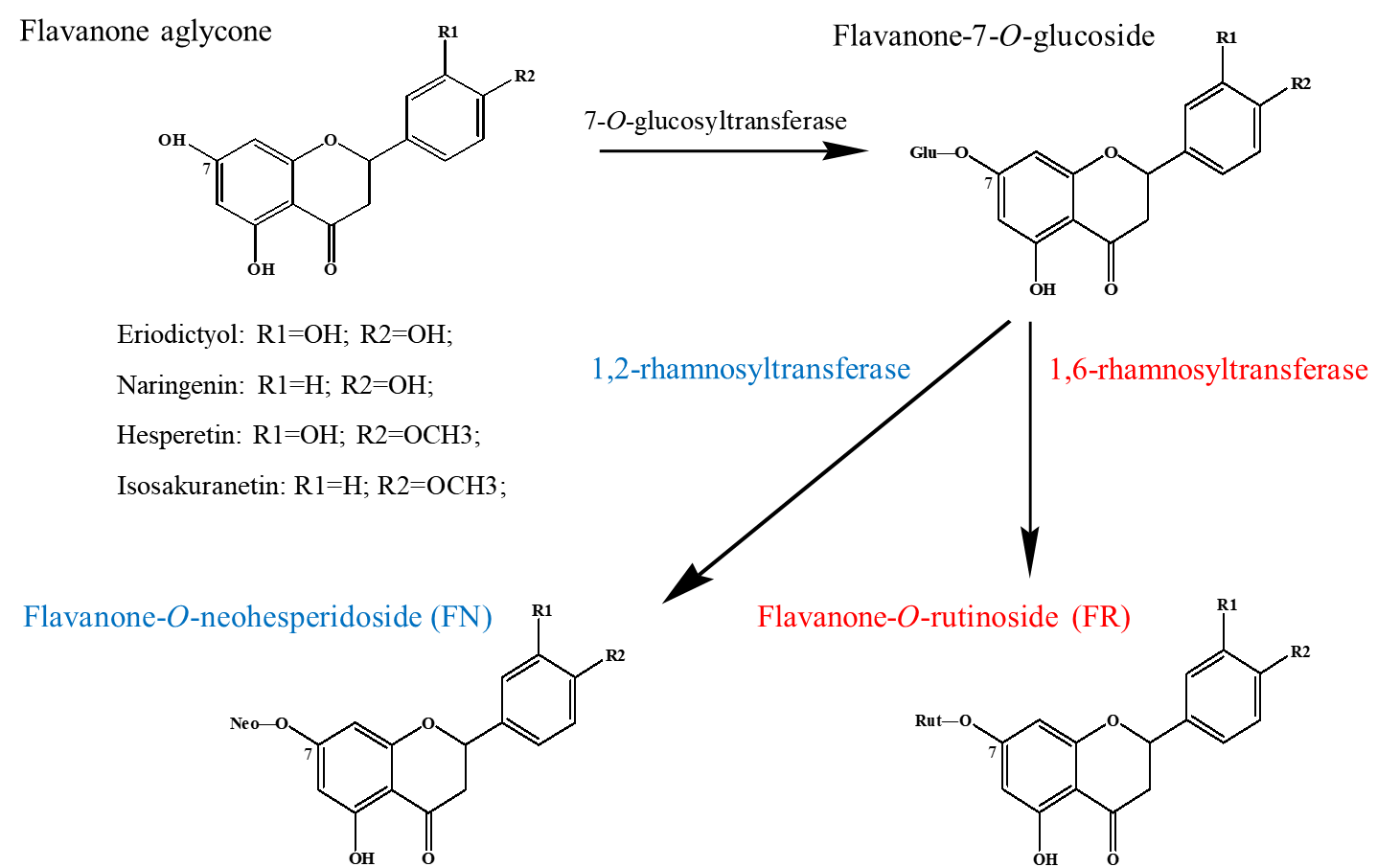


**Fig. S2 Structure and glycosylation process of flavanone glycosides in citrus**.


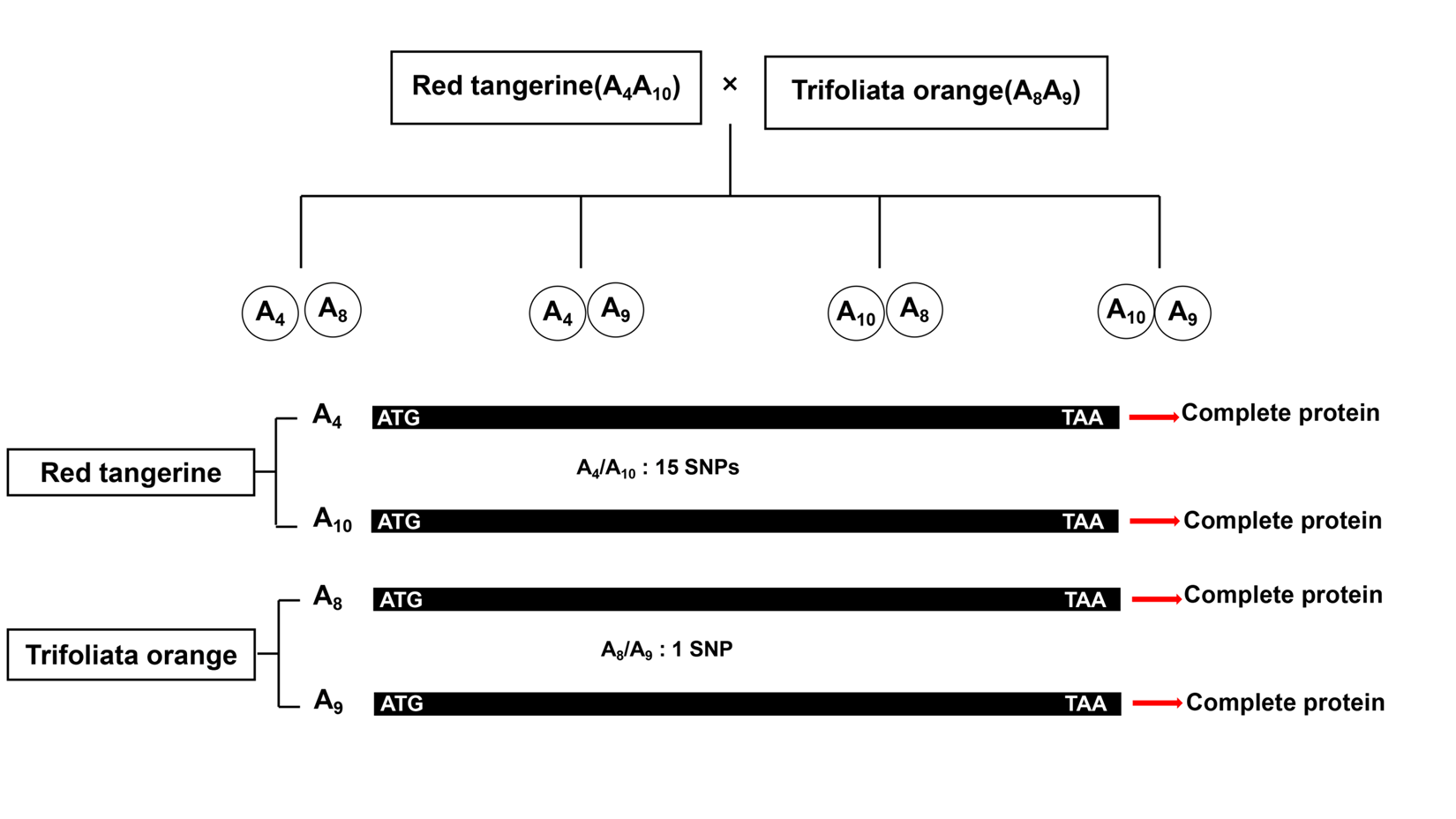


**Fig. S3 Sequence analysis of 1,6RhaT in red tangerine and trifoliate orange and genotype prediction of the F1 progenies.** The nucleotide sequences were in black. ATG, Initiation codon, TAA, Termination codon.


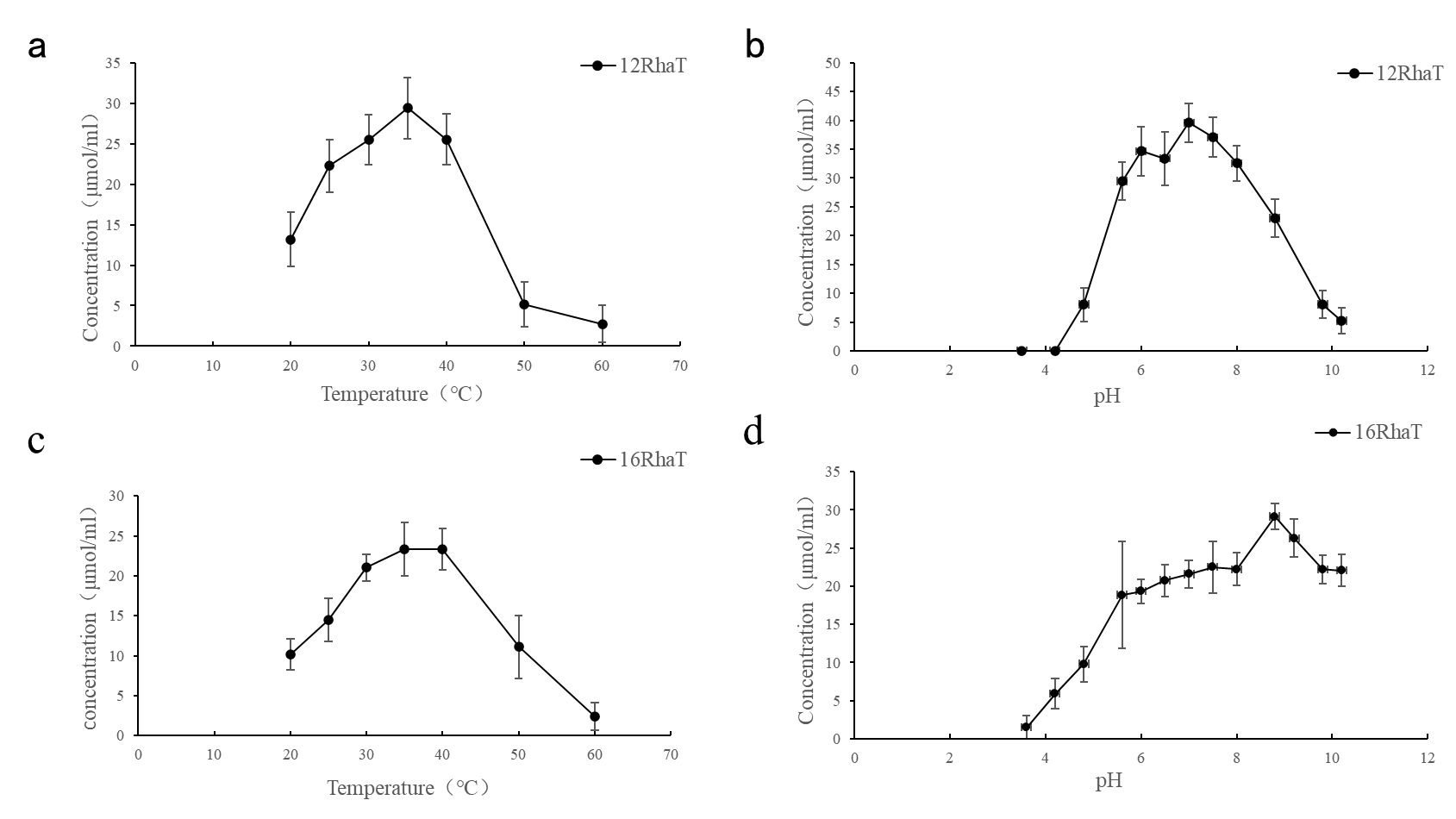


**Fig. S4 Optimization of the reaction pH and temperature for the enzymatic catalysis of recombinant 1,2RhaT and 1,6RhaT.** (**a)** Effect of temperature on the 12RhaT-catalyzed glycosylation of naringenin-7-*O*-glucoside. (**b**) Effect of pH on the 12RhaT-catalyzed glycosylation of naringenin-7-*O*-glucoside. (**c**) Effect of temperature on the 16RhaT-catalyzed glycosylation of naringenin-7-*O*-glucoside. (**d**) Effect of pH on the 16RhaT-catalyzed glycosylation of naringenin-7-*O*-glucoside.


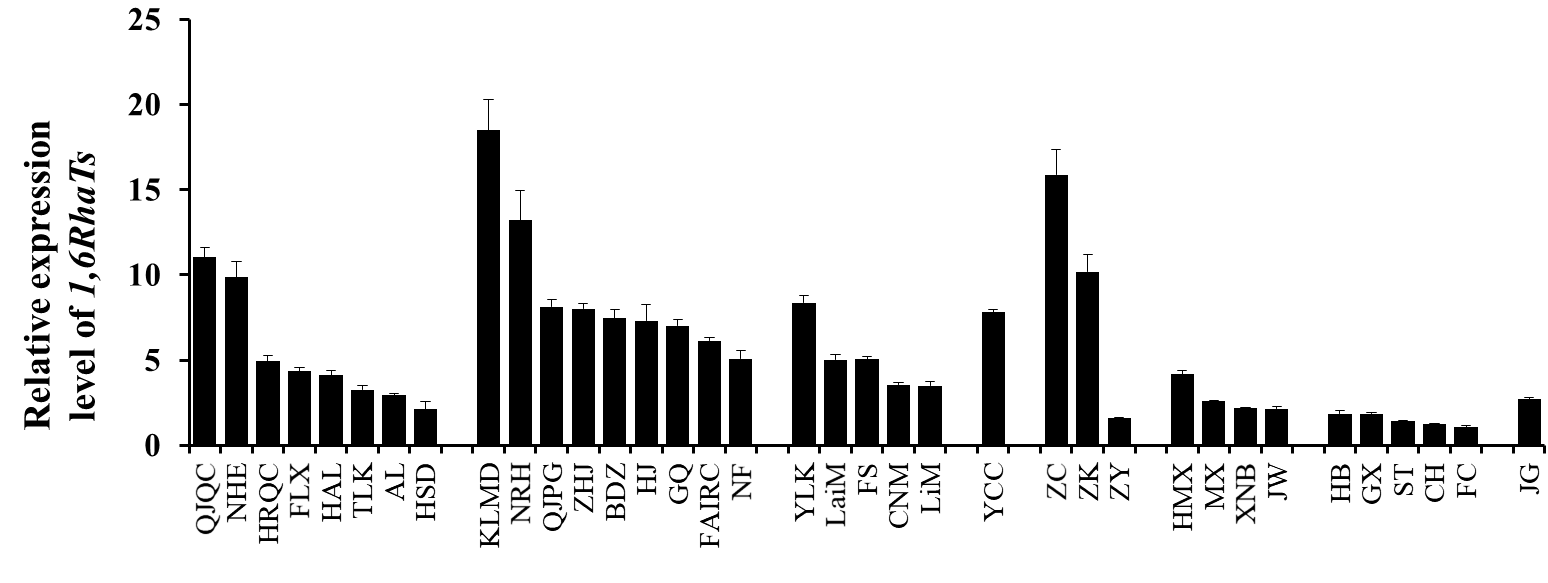


**Fig. S5 Relative expression levels of 1,6RhaT in the fruit peel of various citrus accessions.** Abbreviations of thirty-six citrus accessions were shown in Supplementary Table S1.
